# A systems approach to assess climate change mitigation options in landscapes of the United States forest sector

**DOI:** 10.1186/s13021-018-0100-x

**Published:** 2018-09-04

**Authors:** Alexa J. Dugan, Richard Birdsey, Vanessa S. Mascorro, Michael Magnan, Carolyn E. Smyth, Marcela Olguin, Werner A. Kurz

**Affiliations:** 1 USDA Forest Service, Northern Research Station, 11 Campus Blvd, Suite 200, Newtown Square, PA 19073 USA; 2 USDA Forest Service and Woods Hole Research Center, 149 Woods Hole Road, Falmouth, MA 02540 USA; 3 Consultant to the Commission for Environmental Cooperation, 393 St-Jacques Street West, Suite 200, Montreal, QC H2Y 1N9 Canada; 4 Natural Resources Canada, Canadian Forest Service, 506 Burnside Road West, Victoria, BC V8Z 1M5 Canada

**Keywords:** Forest carbon, Climate change, Mitigation, Greenhouse gas, CBM-CFS3

## Abstract

**Background:**

United States forests can contribute to national strategies for greenhouse gas reductions. The objective of this work was to evaluate forest sector climate change mitigation scenarios from 2018 to 2050 by applying a systems-based approach that accounts for net emissions across four interdependent components: (1) forest ecosystem, (2) land-use change, (3) harvested wood products, and (4) substitution benefits from using wood products and bioenergy. We assessed a range of land management and harvested wood product scenarios for two case studies in the U.S: coastal South Carolina and Northern Wisconsin. We integrated forest inventory and remotely-sensed disturbance data within a modelling framework consisting of a growth-and-yield driven ecosystem carbon model; a harvested wood products model that estimates emissions from commodity production, use and post-consumer treatment; and displacement factors to estimate avoided fossil fuel emissions. We estimated biophysical mitigation potential by comparing net emissions from land management and harvested wood products scenarios with a baseline (‘business as usual’) scenario.

**Results:**

Baseline scenario results showed that the strength of the ecosystem carbon sink has been decreasing in the two sites due to age-related productivity declines and deforestation. Mitigation activities have the potential to lessen or delay the further reduction in the carbon sink. Results of the mitigation analysis indicated that scenarios reducing net forest area loss were most effective in South Carolina, while extending harvest rotations and increasing longer-lived wood products were most effective in Wisconsin. Scenarios aimed at increasing bioenergy use either increased or reduced net emissions within the 32-year analysis timeframe.

**Conclusions:**

It is critical to apply a systems approach to comprehensively assess net emissions from forest sector climate change mitigation scenarios. Although some scenarios produced a benefit by displacing emissions from fossil fuel energy or by substituting wood products for other materials, these benefits can be outweighed by increased carbon emissions in the forest or product systems. Maintaining forests as forests, extending rotations, and shifting commodities to longer-lived products had the strongest mitigation benefits over several decades. Carbon cycle impacts of bioenergy depend on timeframe, feedstocks, and alternative uses of biomass, and cannot be assumed carbon neutral.

**Electronic supplementary material:**

The online version of this article (10.1186/s13021-018-0100-x) contains supplementary material, which is available to authorized users.

## Background

Sustainable forestry activities aimed at maintaining or enhancing carbon (C) stocks in both forest ecosystems and wood products, including product substitution benefits, can make a significant contribution to reducing greenhouse gases (GHG) emissions [43, 45]. To date, the U.S. remains legally committed to reducing net GHG emissions by 26–28% by 2025 compared with 2005 levels [54]. Furthermore the U.S. Mid-Century Strategy set ambitious goals of an economy-wide reduction of GHG emissions of at least 80% below 2005 levels by 2050 [54]. Achieving these goals requires a diverse range of mitigation activities across all economic sectors, including substantial contributions from forests and wood products. Carbon sequestration by U.S. forests currently offsets approximately 15% of annual C emissions from fossil fuels in the U.S. [58] and studies have indicated significant potential to increase this service [37, 41], as well as reduce or delay the expected decline in the forest C sink [58].

**Fig. 1:**
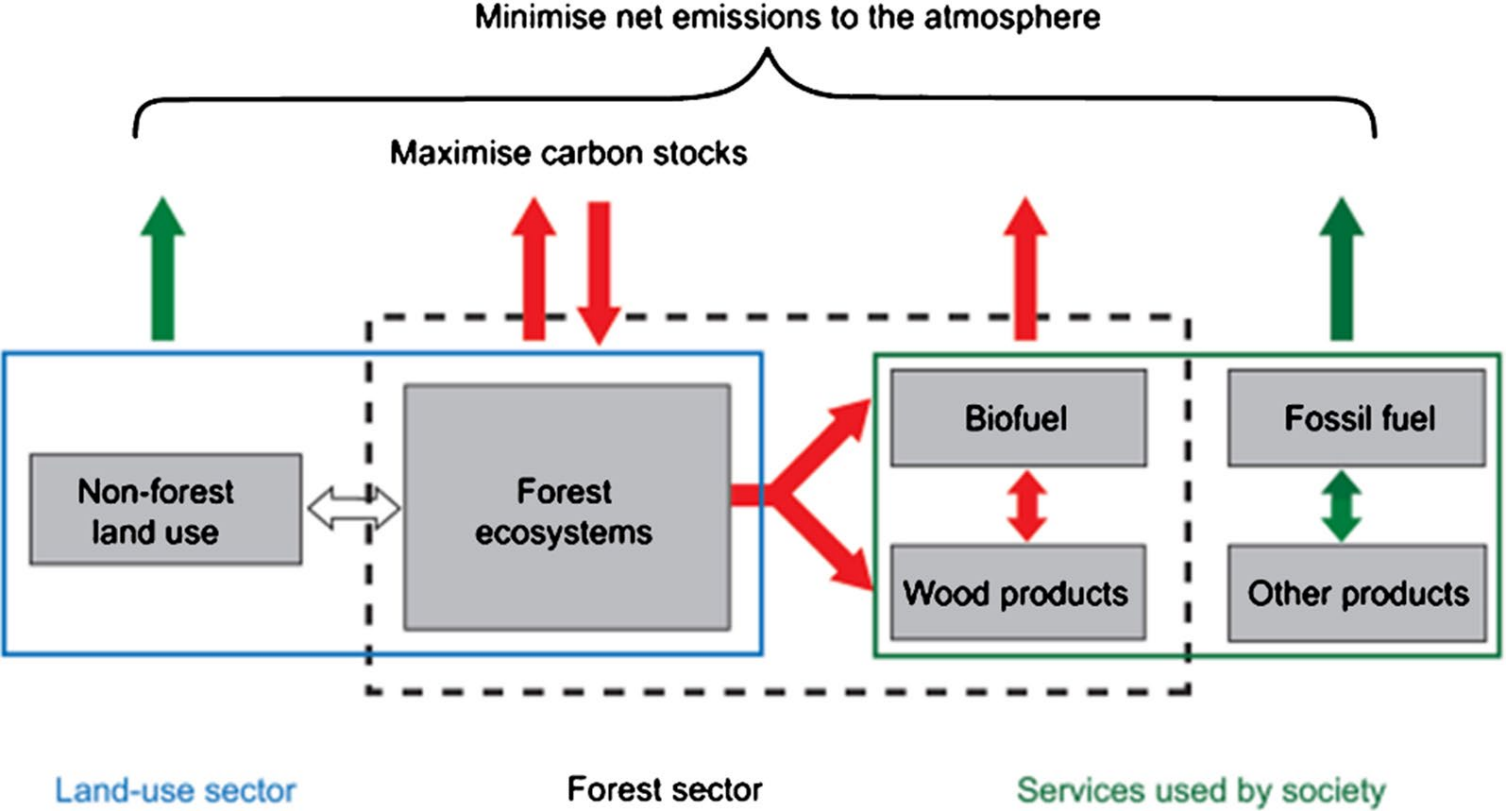
A complete accounting of net forest sector carbon emissions to the atmosphere and mitigation potential requires a systems-based approach which considers the relationships between the forest ecosystem, land use change, harvested wood products, and the substitution benefits associated of using bioenergy (biofuel) and wood products in place of fossil fuel energy and other more emission intensive materials (Graphic reproduced from Nabuurs et al. [45], IPCC Assessment Report 4, Working Group 3, p. 549)

To evaluate the potential contribution of forest sector mitigation, a systems-based approach is necessary, which examines net emissions from four interdependent systems: (1) the forest ecosystem, (2) land-use change, (3) harvested wood products (HWP), and (4) emissions avoided by using wood-based products in place of emission-intensive construction materials and fossil fuels (Fig. 1) [32, 34, 41, 45, 49, 50, 54, 58, 76]. A systems approach accounts for the trade-offs and synergies between mitigation activities that seek to maximize ecosystem carbon stocks and carbon storage in wood products, and minimize emissions by using wood instead of emissions intensive products and fossil energy. If any one component of the forest sector is examined in isolation, it could misrepresent the net emissions to the atmosphere and climate change mitigation potential; hence, the need to examine all simultaneously [32, 45, 48, 54, 58].

The flow of C through forested ecosystems is complex and influenced by many factors including growth and decay rates, disturbance regimes, deforestation, climate change, atmospheric concentrations of CO_2_, and nitrogen deposition [e.g. 50]. Emissions from the wood products sector are determined by the type and life cycles of commodities produced, quantity of mill residues and wastes, decay of retired products in landfills, and life cycle emissions of displaced products [58, 54]. Given these complex and interacting influences on C emissions between forest ecosystems and the products and energy sectors, quantifying the mitigation potential at a landscape scale using an integrated systems approach is essential, but infrequently conducted in the U.S.

This study builds upon previous research that examined modeling approaches to assess past and present changes in forest C stocks at sites in North America [27, 38, 39]. Our principal objective was to use a systems-based approach (Fig. 1) that complies with IPCC and national reporting guidelines to evaluate forest sector climate change mitigation scenarios against projected “business as usual” (BAU) baseline scenarios across two regionally-representative landscapes in the U.S. This study was part of a coordinated tri-national investigation which applied a consistent methodology across a total of six sites in the U.S., Canada [58] and Mexico [49] representing a range of forest ecosystems, climates, and disturbances, and management regimes. The data and methods also build upon past studies which assessed historical baseline ecosystem C stocks and influences across the U.S. National Forest System [6, 17, 50]. We expand the previous research to include analyses of projected C dynamics across two, multi-ownership landscapes and by integrating accounting of HWPs and substitution benefits. In consultation with stakeholders from each site, we evaluated a limited set of site-specific scenarios that represent practical ways to sustainably manage forests to reduce GHG emissions while minimizing tradeoffs with other ecosystem services and if possible, increasing production of forest commodities.

## Methods

### Study areas

**Fig. 2:**
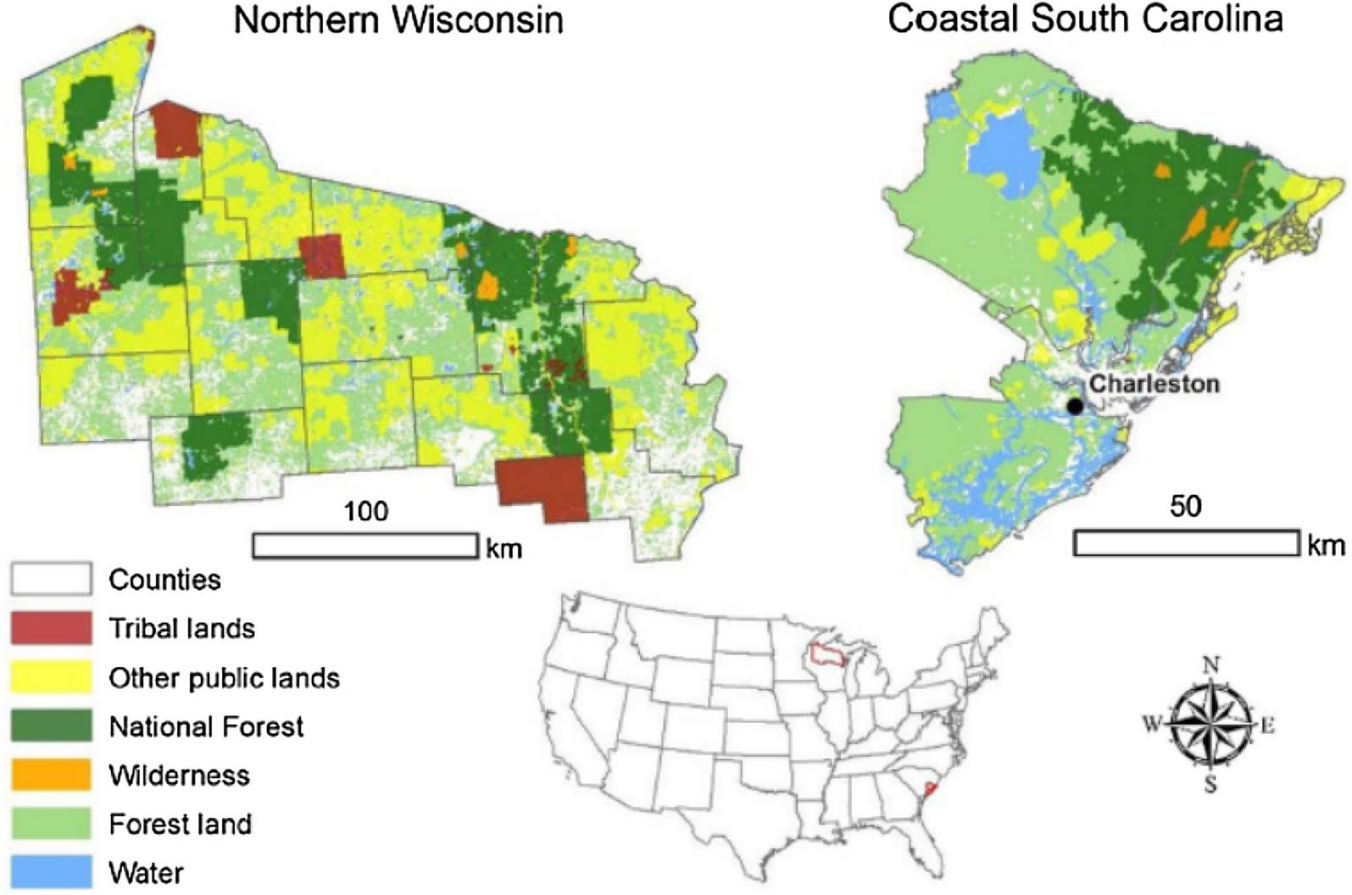
The coastal South Carolina and Northern Wisconsin study areas. Areas not coded as other public, tribal, or national forest are privately owned

**Fig. 3:**
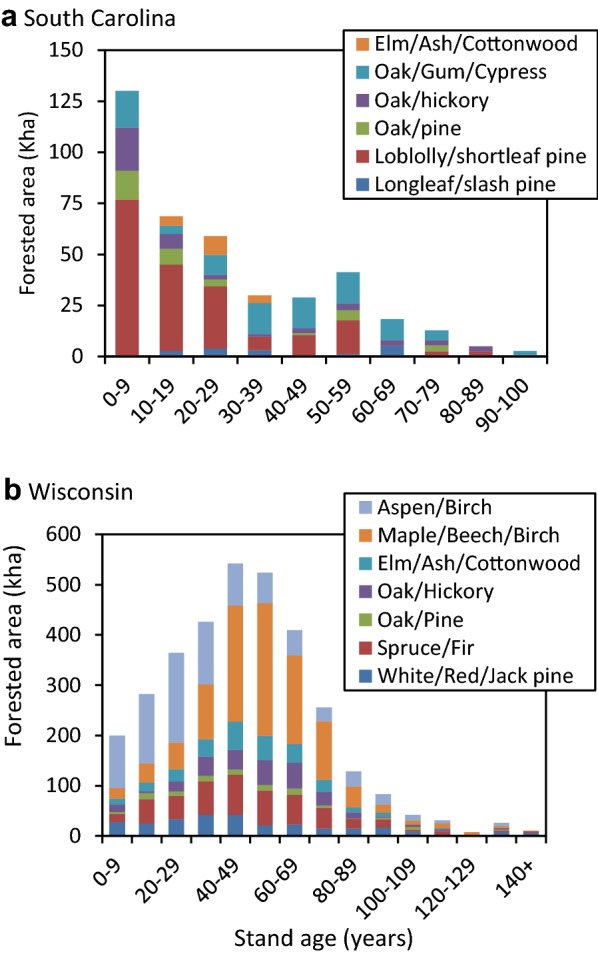
Stand age distributions in 1990 by forest type groups for the **a** coastal South Carolina and **b** Northern Wisconsin study areas

In consultation with study sponsors and companion studies in Canada [58] and Mexico [49], we selected two study sites to serve as case studies, Coastal South Carolina (SC) and northern Wisconsin (WI). Both sites are strongly oriented around the timber industry and thus have significant opportunities to implement changes in land management activities and the HWP sector. Each site represents a heterogeneous, multi-ownership landscape (Fig. 2) with a range of management regimes, disturbance histories, and stand-age structures. Forests in both study areas are affected by a history of intensive harvesting and reversion of abandoned agricultural land to forest in the early 1900s, followed by a period of recovery, forest management, and restoration [4]. In the Northern WI site, the legacy effects of past land use are evident in the age-class distribution (Fig. 3) and declining growth rates of stands that continue to age without recent disturbance. In contrast, forests in coastal SC are recovering from the destructive 1989 Hurricane Hugo [54], resulting in younger, more productive forests at the start of the simulation (Fig. 3). The coastal SC site has experienced substantial deforestation, especially on private lands, due to rapid development and population growth in Charleston, SC in recent years [58], while urbanization pressures are smaller in Northern WI.

### Data and models

To assess net GHG emissions and mitigation potential in forest ecosystems and the land-use sector, we employed a spatially-referenced approach with the Carbon Budget Model for the Canadian Forest Sector (CBM-CFS3). This is a growth-and-yield driven model that employs a gain–loss method consistent with the IPCC reporting guidelines [22]. CBM-CFS3 quantifies the cycling of C through the ecosystem. The model incorporates (1) forest inventory data to stratify stands by classifiers such as forest types, ownerships, stand origin and stand ages, (2) estimates of historical disturbances, harvests, and land-use change to target activities to specific stands and areas using the classifiers and eligibility rules, and (3) growth and yield curves to model growth rates and recovery. See Kull et al. [27], Kurz et al. [27], and Additional file 1 for details on CBM-CFS3 and data inputs. We also compared historical forest C stocks derived from the CBM-CFS3 to other empirical and process model results [6, 58] to validate CBM-CFS3 baseline results and to infer the potential C impacts of climate and atmospheric conditions.

To assess the mitigation potential in the products sector, C transferred from the forest to products was tracked with the Carbon Budget Modelling Framework for Harvested Wood Products (CBMF-HWP) [54, 58, 76]. The model estimates emissions of harvested C through the lifecycle of manufactured commodities and includes burning of bioenergy, mill residue use and waste, exports, and post-consumer treatment (landfill and/or waste incineration) (see Additional files 1, 2).

We compiled site-specific model input data for the historical period of 1990 to 2011—the last year for which data were uniformly available. We obtained information on forest characteristics such as ownership, forest type, stand age (Fig. 3), and growth and yield curves (e.g. Additional file 1: Figure S1) from the Forest Inventory and Analysis (FIA) database and tools [74, 75]. We determined annual area disturbed from Landsat satellite imagery-based disturbance maps [12, 17] and followed the approach of Mascorro et al. [38, 39] to attribute the causes of disturbances to fire, insects, and abiotic factors using ancillary data. We used historical land-cover change estimates from the National Land Cover Database (NLCD) [9, 18, 19] as a proxy for land-use change. We utilized the FIA Timber Product Output (TPO) database to determine the volume of timber removals by ownership on public and private lands. We obtained data on product ratios (e.g. pulpwood, saw logs, composites, etc.) (Additional file 1: Figure S5) and production and export of HWP from periodic regional timber industry assessments (e.g. 26, [15]) and national statistics [20, 23]. Product half-lives for each commodity were based on values from Skog [58] and the IPCC [23]. We applied average annual land-use change, disturbances rates, and commodity production data from 2002 to 2011 to the projection period baseline (2012–2050).

Displaced emissions, defined as the difference in the amount of C emitted if alternative fuel sources or products had instead been utilized [50], [54], were also included in the systems-approach. Displacement factors were used to describe the avoided fossil carbon emissions per unit of wood carbon used. We applied the following average displacement factors, calculated at the national level for Canada [54]: 0.54 tC displaced per tC of saw and veneer logs, 0.45 tC displaced per tC of panels, and 0.89 tC displaced per tC of bioenergy. Our study applied similar system boundaries and end-use products as Smyth et al. [54]. Displacement factors for wood substitution were based on the relative emissions from a more wood-intensive product versus a less wood-intensive product for general end-uses of wood (homes, multiuse building, furniture, flooring, and decking) and included emissions associated with extraction and transportation of raw materials and manufacturing. Displacement factors for bioenergy substitution assume that only energy from fossil fuel sources were displaced and were derived by comparing bioenergy facility emission-intensities to those for the production of energy from fossil fuels for heat, electricity or combined heat and power. To calculate the avoided emissions for each product type, displacement factors were multiplied by the increase or decrease in wood products or bioenergy [54, 58, 76].

### Mitigation scenarios

Based on consultation with stakeholders who were knowledgeable about regional forest practices and conditions represented by the study sites, we selected eight mitigation scenarios for the coastal SC site (Table 1) and five for the northern WI site (Table 2). Scenarios targeted individual ownerships, and typical forest management activities and/or product ratios.

**Table 1:** Indicators for the eight mitigation scenarios for the coastal South Carolina site

Scenario	Description	Parameter changed^a^	Parameter value^a^
Residues	Increase collection of harvest residues for bioenergy^b^	Residues recovered (%) HWP component changes	40% to 70% Bioenergy + 30%
Productivity^c^	Increase productivity of half of existing, loblolly pine plantations through silvicultural activities	Additional disturbance type Proportion targeted Increase growth curve	Increase productivity 50%/year 15% increase
Reduce deforestation^c^	Reduce annual area deforested on private land	Deforestation rate (area)	25% reduction/year (1304 to 978 ha/year)
No net loss^c^	Increase annual area afforested to equal deforestation rate on private land	Afforestation rate (area)	3 × increase (432 to 1304 ha/year)
Longer-lived products (LLP)	Increase the proportion of harvested wood for LLP at the cost of paper products (PP)	HWP components change	LLP + 10%, PP − 10%
Bioenergy	Increase the proportion of harvested wood for bioenergy at the cost of LLP	HWP components change	Bioenergy + 10%, LLP − 10%
Hurricane—Hugo salvage^d^	Simulate a hurricane in 2018 with effects and salvage rates mimicking Hurricane Hugo	Percentage of wood salvaged	SW: 0% to 14% HW: 0% to 1.2% (of mortality)^e^
Hurricane—Increase salvage^d^	Simulate a hurricane in 2018 with effects mimicking Hurricane Hugo, but increase salvage rates	Percentage of wood salvaged	SW: 0% to 31% HW: 0% to 5% (of mortality)^e^

^a^The parameter changes are relative to the baseline scenario

^b^Residues would otherwise decompose on forest floor

^c^Private lands only

^d^Evaluated against a hurricane baseline scenario which assumes no salvage logging

^e^Salvage rates are a percentage of the hurricane-induced mortality

**Table 2:** Indicators for the five mitigation scenarios for the Northern Wisconsin site

Scenario	Description	Parameter changed^a^	Parameter value^a^
Residues	Increase collection of harvest residues for bioenergy^b^	Residue utilization HWP components change	29% to 70% Bioenergy + 41%
Harvests for bioenergy	Increase harvests by 10% per year, with 100% of the additional harvested wood used for bioenergy	Harvested area HWP components change	+ 10% 100% to bioenergy (added harvests)
Extend rotation + Longer-lived products (LLP)	Extend the length of harvest rotation and increase the proportion of LLP at the cost of paper products (PP)	Harvested area Minimum harvest age HWP components change	− 10% +10 years LLP + 5%, PP − 5%
LLP	Increase the proportion of harvested wood for LLP at the cost of PP	HWP components change	LLP + 10%, PP − 10%
Bioenergy	Increase the proportion of harvested wood for bioenergy at the cost of LLP	HWP components change	Bioenergy + 10% LLP − 10%

^a^The parameter changes are relative to the baseline scenario

^b^Residues would otherwise decompose on forest floor

Total emissions for each scenario were calculated as the sum of (1) net forest ecosystem emissions due to carbon removals from the forest, on-site decay of harvest residues, and disturbance and land-use change impacts, (2) HWP emissions from commodity production, product use (including bioenergy burning), export, and decomposition from mill wastes and post-consumer use, and (3) changes in emissions due to substitution of bioenergy for fossil fuels and solid-wood products for more emission-intensive materials. The cumulative mitigation effect of each scenario was the difference between net GHG emissions (CO_2_, CH_4_, CO, N_2_O) of the mitigation scenario minus net GHG emissions of the baseline scenario, which isolates the effects of the targeted mitigation parameters while factoring out any effect common to the scenarios (“additionality”). We assumed stable CO_2_ levels and climate throughout the simulation period for the baseline and all scenarios so that these effects would be factored out when estimating the additional changes in GHG emissions.

More thorough descriptions of the data inputs, model parameters, and mitigation scenarios are available in Additional files 1, 2.

## Results

### Historical and baseline emissions

**Fig. 4:**
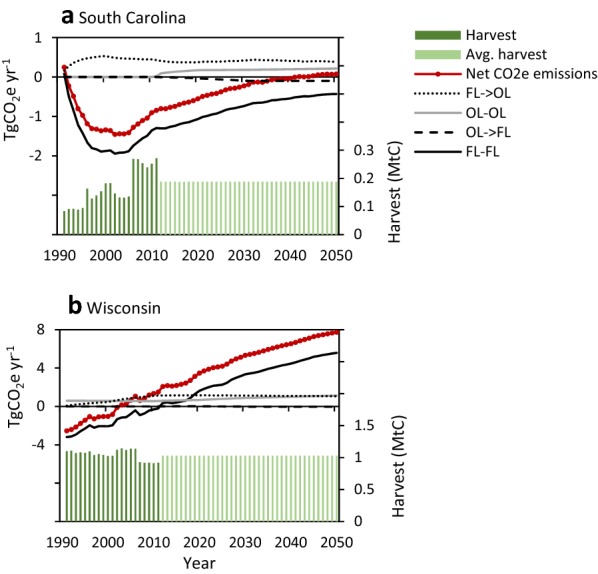
Time series of the annual GHG emissions (left axis) from each land class within the forest ecosystem including: forest land remaining forest (FLFL), other (nonforest) land remaining other (OLOL), forest converted to other (FL → OL), other converted to forest (OL → FL), and all land classes combined (Net CO_2_e) from 1990 to 2050 for the **a** coastal South Carolina and **b** Northern Wisconsin study areas. Positive values indicate a release of GHGs to the atmosphere (carbon source). The historical harvest (MtC) per year are shown by the dark green bars and the 10-year average (2002–2011) harvest is shown by the light green bars (right axis)

**Fig. 5:**
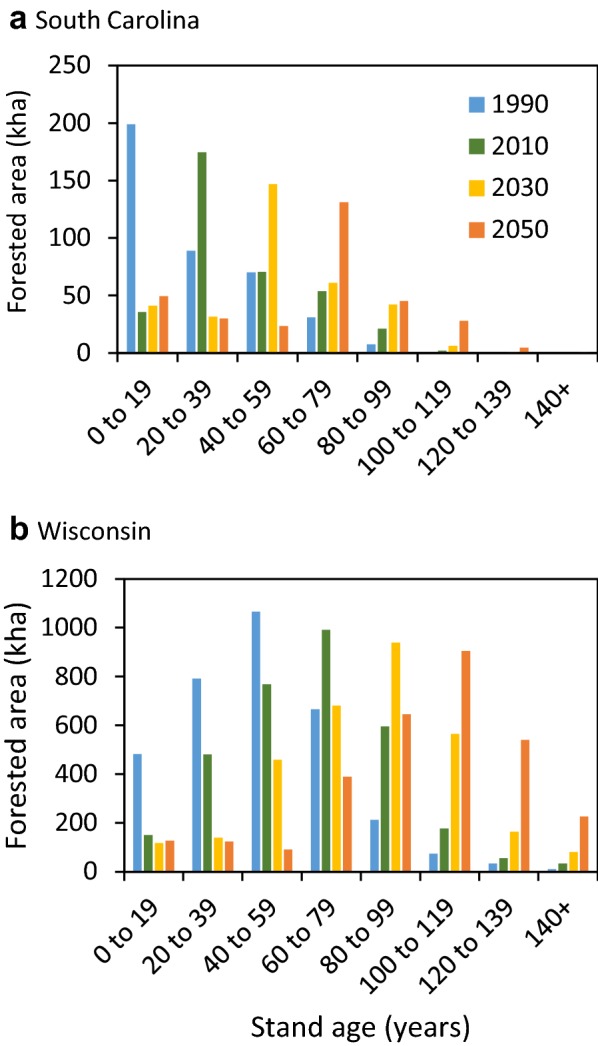
Age structure time series from 1990 to 2050 for the **a** coastal South Carolina and **b** Northern Wisconsin study areas

Within the forest ecosystem, the baseline for the coastal SC study site remained a net C sink from 1991 through 2042 before switching to a source through 2050 (Fig. 4a). The C sink is strongly influenced by regrowth and recovery following Hurricane Hugo in 1989, reflected in the younger age-class structure in the 1990s (Fig. 3a). A sharp increase in timber harvesting on private lands beginning in 2006 increased C emissions. As more stands reached an older growth stage (Fig. 5a), mortality increased while productivity declined or stabilized causing the C sink to decline further through 2042 when the landscape is projected to become a source of C emissions (Fig. 4a). To a lesser extent, the projected increase in GHG emissions results from a decline in forested area due to continued net deforestation (Additional file 1: Figure S6) of roughly 0.2%/year which occurs mostly on private lands (Additional file 1: Table S3). Over the historical period, ecosystem C stocks on forested lands increased from roughly 169 tC ha^−1^ in 1990 to 175 tC ha^−1^ in 2016 with National Forest lands experiencing the largest increase. These modeling results for the baseline were validated and consistent with an inventory-based stock-change model and an independent process-based model (Additional file 1: Figure S7).

The net C balance of the forest ecosystem of the northern WI site shows a steady increase in GHG emissions causing a shift from a C sink to a source in 2022 (Fig. 4b). Like coastal SC, this projected shift to a C source is largely a result of forest aging (Fig. 5b) causing net volume and biomass accumulation to approach zero (Additional file 1: Figure S1). This aging effect was coupled with a decline in forest area from deforestation of 0.1%/year. However, over the historical period, forest ecosystem C stocks across all ownerships were relatively stable, a trend that is consistent with inventory data and the process model (Additional file 1: Figure S8). The process model found that increasing CO_2_ has caused significant increases in C accumulation over the past few decades for both study sites (Additional file 1: Figure S9).

### Results of mitigation scenarios

#### South Carolina

**Fig. 6:**
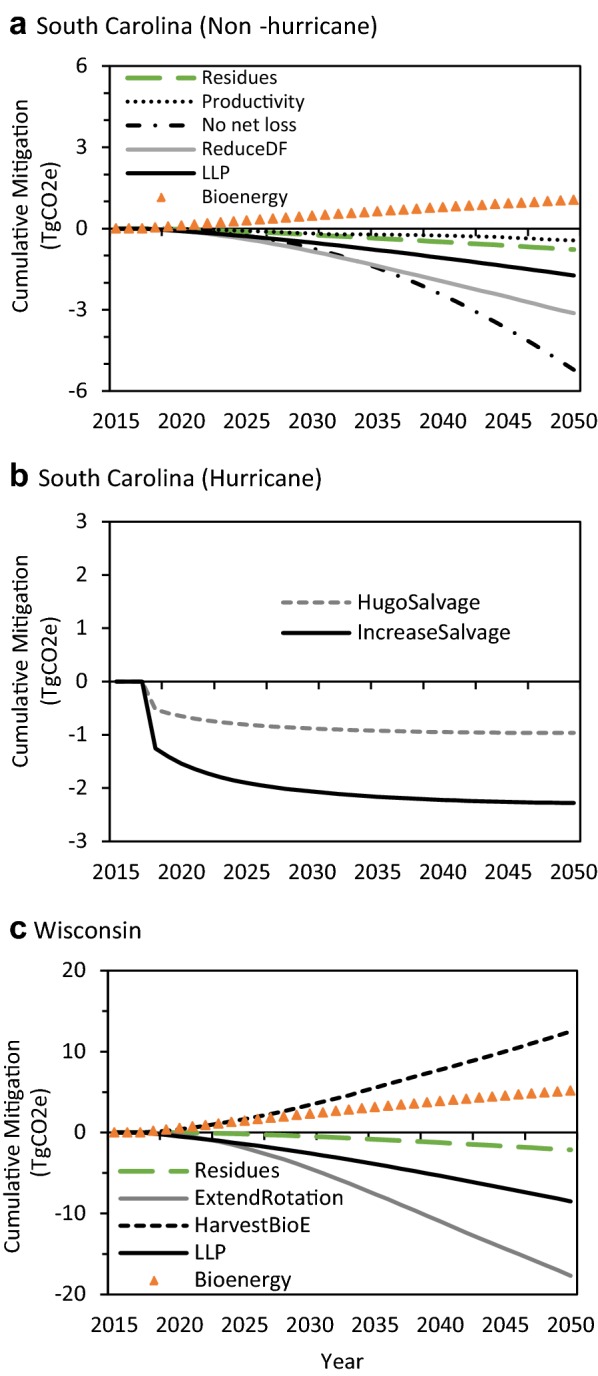
Time series of the total cumulative mitigation relative to the baseline for each scenario for **a** coastal South Carolina (non-hurricane scenarios), **b** coastal South Carolina (hurricane scenarios), and **c** Northern Wisconsin study areas. Negative values denote a reduction in GHG emissions

**Fig. 7:**
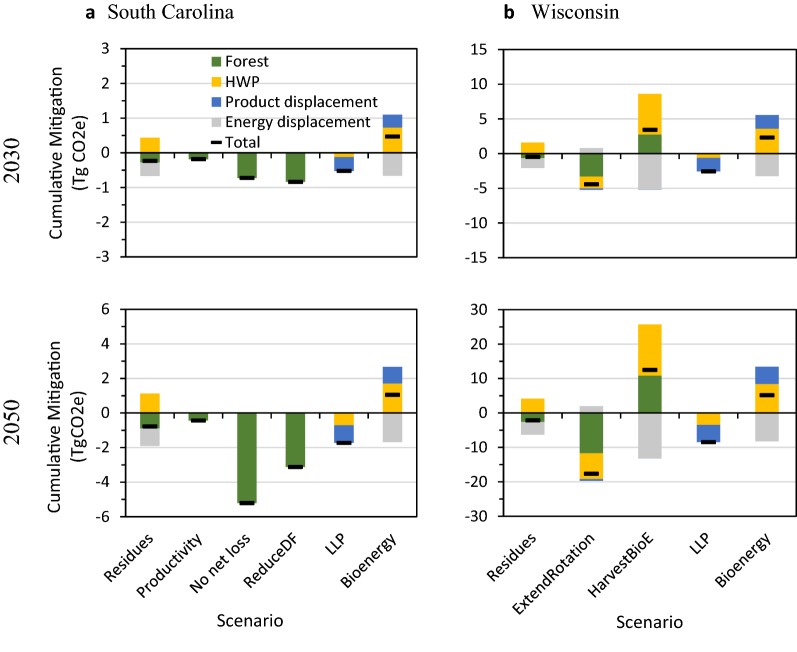
Cumulative mitigation by component in 2030 and 2050 for the **a** coastal South Carolina and **b** Northern Wisconsin study areas. Negative values denote a reduction in GHG emissions

The two scenarios targeting land-use change on private lands had the greatest mitigation benefit: the *no net loss* scenario represents the effect of increasing afforestation and resulted in a cumulative net reduction of 5.2 Tg CO_2_e by 2050. The *reduce deforestation* scenario had a net reduction of 3.1 Tg CO_2_e by 2050 (Figs. 6a, 7a) and ranked first until 2033, but then was surpassed by the *no net loss* scenario as the annual mitigation increment increased substantially in the 2030s and 2040s (Table 3). The *no net loss* scenario resulted in a three-fold increase in the rate of afforestation to over 1300 ha per year on private lands, whereas reducing deforestation on private lands by 25% per year still resulted in a net loss in forest land annually (Additional file 1: Figure S6).

**Table 3:** Average annual mitigation (in Tg CO_2_e year-1) for each decadal range for the mitigation scenarios in the two study regions

Scenario	2021 to 2030^a^	2031 to 2040^a^	2041 to 2050^a^
Coastal South Carolina			
Residues	− 0.022	− 0.026	− 0.028
Productivity	− 0.017	− 0.006	− 0.018
No net loss	− 0.072	− 0.179	− 0.280
Reduce deforestation	− 0.079	− 0.112	− 0.118
LLP	− 0.043	− 0.057	− 0.064
Bioenergy	0.036	0.032	0.026
Hugo salvage^b^	− 0.023	− 0.007	− 0.002
Increase salvage^b^	− 0.053	− 0.016	− 0.005
Northern Wisconsin			
Residues	− 0.047	− 0.077	− 0.090
Extend rotation + LLP	− 0.409	− 0.656	− 0.670
Harvest bioenergy	0.301	0.436	0.473
LLP	− 0.210	− 0.278	− 0.314
Bioenergy	0.176	0.156	0.130

^a^Negative values indicate a reduction in CO_2_e emissions

^b^Evaluated against a hurricane baseline scenario which assumes no salvage logging

The two forest management scenarios also had mitigation benefits, though smaller than the land-use change scenarios. The increased use of logging *residues* for bioenergy scenario caused a reduction in net emissions of 0.8 Tg CO_2_e by 2050 (Figs. 6a, 7a). This was a result of reduced emissions from the forest ecosystem because, after timber extraction, woody residues were transferred to the HWP sector and burned in a bioenergy facility, rather than left on-site to decay over time, plus substitution benefits from avoided fossil fuel burning. Increasing the *productivity* of loblolly pine plantations had a small positive cumulative mitigation benefit on the forest ecosystem of 0.4 Tg CO_2_e, but no effect on HWP or displacement, because harvest rates remained constant in this scenario.

The increase in *longer*-*lived products* (*LLP*) scenario ranked third, with a cumulative mitigation of 1.7 Tg CO_2_e due to a reduction in emissions from HWP because of longer product half-lives and increased avoided emissions from using more saw and veneer logs relative to the baseline (Figs. 6a, 7a). Despite displacing emissions from fossil fuel use, the *bioenergy* scenario, which increased the proportion of bioenergy by 10% at the cost of LLP, increased emissions by roughly 1.1 Tg CO_2_e. This increase in emissions was largely a result of greater emissions from the HWP component by shifting LLP to bioenergy, which causes immediate emissions, and resulted in a negative product displacement effect.

The two hurricane scenarios, *Hugo salvage* and *increase salvage*, which salvaged dead wood at varying rates after a hypothetical hurricane in 2018, had positive mitigation benefits relative to the no salvage baseline (Fig. 6b, Additional file 1: Figure S10). Both scenarios resulted in fewer forest ecosystem emissions as less dead wood was left on-site to decay, which were counteracted by a comparable increase in HWP emissions due to more wood being processed and utilized (Additional file 1: Figure S10). Thus the mitigation benefit was mostly due to salvaged wood displacing both emission-intensive fuels and building materials. The largest average annual mitigation benefit of salvage logging occurred in the year of the hurricane, and was reduced thereafter (Fig. 6b; Table 3).

Several scenarios could be assessed together since they involve different parts of the land base and therefore do not interact. For example, the combined effects of reducing deforestation while increasing afforestation (*no net loss*), use of logging *residues*, *productivity*, and *LLP* had a combined cumulative mitigation benefit of 11.3 Tg CO_2_e through 2050.

#### Northern Wisconsin

The *extend rotation *+* LLP* scenario ranked first, reducing cumulative net emissions by 17.7 Tg CO_2_e by 2050, due to its strong mitigation benefit in both the forest ecosystem and HWP components (Figs. 6c, 7b). Reducing harvesting by 10% caused more live biomass to remain in the forest to sequester CO_2_ and reduced HWP emissions. By shifting an additional 5% of the products from pulp and paper to LLP, the scenario had a small product displacement benefit; however, less harvested wood also meant less bioenergy and therefore less substitution benefits from avoiding fossil fuel burning. Increasing the proportion of *LLP* without extending the rotation length ranked second, reducing net emissions by 8.5 Tg CO_2_e by 2050 (Figs. 6c, 7b). Although this scenario had no effect on the forest, it reduced HWP emissions by increasing product lifespans thereby delaying C release relative to the baseline, and avoiding emissions intensive materials (Fig. 7b).

The three bioenergy-related scenarios ranked lowest and two of them increased emissions relative to the baseline (Figs. 6c, 7b). As in SC, increasing use of logging *residues* for bioenergy had a mitigation benefit (2.1 Tg CO_2_e by 2050) by reducing ecosystem emissions and increasing substitution for fossil fuels, which outweighed the negative effect on HWP given higher emissions from additional bioenergy. Also like the SC site, shifting utilization from LLP to *bioenergy* had a negative effect on HWP as it resulted in more immediate emissions, which were not completely compensated by a reduction in fossil fuel use. The increase *harvest for bioenergy* scenario ranked last, causing a cumulative increase in net emissions of 12.5 Tg CO_2_e relative to the baseline by 2050. Despite having a strong energy displacement effect, increasing harvests for bioenergy was not able to offset increased emissions and lost productivity from more trees being harvested and increased instantaneous oxidation from biomass burning (Fig. 7b).

As in the SC site, some scenarios in the WI site could be implemented together. For instance, using additional logging *residues* for bioenergy while also shifting commodities to use more *LLP* had a combined net mitigation benefit of 10.6 Tg CO_2_e through 2050.

## Discussion

### Forest trends and mitigation

Results from these two landscapes suggest that the net C sink in managed forests of the Eastern U.S. is projected to decline under BAU scenarios. Forests in the Northern WI site may soon become a C source (Fig. 4), but those in the coastal SC site will remain a C sink for several decades, even with increased harvest, because the forest is still young and productive as it recovers from the 1989 hurricane. Over much of the 20th century and continuing today, forests in the Eastern U.S. have been net C sinks due to recovery after heavy logging and clearing for agriculture from the mid-1800s to early-1900s [4]. The recent increase in forest ecosystem C emissions and reduced sink is largely due to forests aging (Fig. 5) and becoming less productive [e.g. 3, 16] as depicted by the sigmoidal growth-and-yield curves that drive biomass C accumulation in the model (Additional file 1: Figure S1); [27]. This trend has been documented across many U.S. national forests [6] and is projected to continue through the 21st century, potentially resulting in a national-scale shift from a C sink to a C source [4, 58]. Although yield curves indicate that biomass C stocks may be approaching maximum levels, ecosystem C stocks can continue to increase for many decades as dead organic matter and soil C stocks continue to accumulate. Several mitigation scenarios modeled here could reduce net C emissions while others could increase emissions.

The expected decrease in the net C sink of the SC site is also driven by a decline in forested area as a result of continuing deforestation, notably around rapidly growing metropolitan areas like Charleston, SC. If current rates of forest loss continue, the coastal SC site may lose nearly 7% of its forest area by 2050. In the SC site, the *no net loss* and *reduce deforestation* scenarios that targeted private lands, have significantly higher mitigation potential than all other scenarios evaluated (Fig. 7a). While the *no net loss* scenario resulted in the greatest reduction in C emissions by 2050, reducing deforestation by 25% per year would significantly reduce emissions, have a more immediate mitigation benefit, and be relatively cheaper than a threefold increase in reforestation (Additional file 1: Figure S6) [7]. Activities that maintain or expand forests are often the most effective biophysical mitigation strategies involving forests, and have considerable ecological co-benefits [13, 25, 49, 50].

Other effective scenarios include those that maintain growing stock and shift the commodity mix to longer product lifetimes. In WI, the *extend rotation *+* LLP* scenario which combines both forest management and HWP actions had the largest C benefit. Extending rotations results in more growing stock left in forests to sequester C and a decrease in emissions from product use and post-consumer treatment. However this scenario has tradeoffs in that it reduces substitution benefits due to less timber supply [2, 35, 47]. Likewise, maintaining baseline harvest levels but shifting commodities from shorter-lived to longer-lived products were effective scenarios in both sites. Results here agree with those in Canada [54, 58] and Europe [14, 36, 76] which found that HWP scenarios that increase product retention time outperform those that increase bioenergy.

Enhancing bioenergy production generally led to increases in C emissions over this timeframe except for scenarios that collected additional logging residues for bioenergy. The *residues* scenarios had modest mitigation benefits consistent with results of other studies [10, 11, 58, 76]. If considering increasing the use of logging residues, sustainable management guidelines suggest leaving at least 30% of residues on-site [10, 11, 54] since complete removal of residues could have tradeoffs including a decline in soil nutrients and long-term site productivity [1, 58, 76]. None of our results supported the notion that using wood for bioenergy is “carbon neutral.” Rather, our study shows that over this 32 years period increasing harvests or allocating more harvested wood for bioenergy does not result in a sufficient substitution benefit to compensate for the increase in emissions from the immediate combustion of biomass or the reduction in ecosystem C stocks and uptake, as described elsewhere [21, 40, 54, 54].

Climate change may cause more frequent and intense hurricanes, which can reduce long-term C storage in forests [42]. Results for SC suggest that if another Hurricane Hugo were to hit the coastal U.S., increasing salvage rates above those implemented after the 1989 hurricane would cause a substantial reduction in CO_2_e emissions because the salvaged wood displaces emissions from non-woody building materials and fossil fuels, rather than decomposing in the forest.

### Limitations

We did not analyze potential spatial or temporal leakage—the risk that emissions may be displaced outside of the project boundaries resulting in a diminished mitigation benefit. For example, if harvest rotations are extended, demand for wood may shift elsewhere, which could be significant locally or regionally though insignificant at national or global scales given the size of our study landscapes. When evaluating mitigation scenarios it is important to address macro-economic factors driving supply and demand for products, and broader impacts on land use, both of which may have significant effects on the results and feasibility [e.g. 31, 46, 76].

Another limitation is the relatively short timeframe of our analysis. Other studies have shown that mitigation benefits may vary considerably over longer periods of time since the effect of CO_2_ removal by forest ecosystems and transfer to wood products pools including displacement continues to accumulate compared with a baseline that eventually ceases to accumulate C in the forest ecosystem [44, 54]. For example, our results appear unfavorable for increasing harvest for bioenergy over several decades, but if the analysis were extended to 50 or more years, mitigation benefits are likely to be achieved as forests would have had time to recover. However, long-term analyses introduce other uncertainties resulting from the unknown impacts of climate change, natural disturbances, and socioeconomic factors, such as the reduction in fossil fuel energy displaced. Furthermore, evaluation of short-term mitigation potential is needed considering it will be necessary to achieve net zero global emissions by roughly 2060 if the goal is to limit warming to 1.5–2 °C by the end of the century [24].

Uncertainties also exist in modeling assumptions, parameters, and datasets. For instance, we utilized average displacement factors developed at the national-scale in Canada [54]. Bioenergy displacement factors are dependent on region-specific variables including population, road networks and accessibility to forests, consumption patterns, and energy demand. However, the relatively high bioenergy displacement factor applied (0.89 tC/tC) allowed us to evaluate a high potential energy displacement for which a mix of coal, fuel oil and natural gas are assumed to be avoided. Also, the production displacement factors developed for general wood use in Canada may under-represent the avoided emissions from using wood products in place of steel, concrete and plastic, given the higher displacement factors reported in the meta-analysis by Sathre and O’Connor [54]. However, as other studies have noted, the average value from the meta-analysis included a variety of system boundaries, which produced a large range of displacement factors [5, 54, 58]. Further research on bioenergy and product displacement factors in the U.S. is warranted, and additional consideration given on how to best direct incremental harvest products to avoid emissions-intensive products and fossil fuels.

Using a land-cover change product to approximate land-use change may have resulted in an overestimation of the area of land-use change, thus impacting the baseline scenario [38]. However, this potential overestimation would not have significantly impacted the evaluation of mitigation scenarios because land-use change and disturbance rates were applied equally in both the baseline and mitigation scenarios, which isolates the impacts of mitigation actions (additionality). Even for mitigation scenarios targeting land-use change or harvest, we generally evaluated percentage increases or decreases in parameters (Table 2), thus baseline areas of land-use change and disturbance would have little impact on the evaluation of these mitigation scenarios. Nationally-consistent remote sensing products that attribute land-cover changes to land-use conversions, disturbances, and management activities on an annual basis would greatly improve the ability to estimate ecosystem carbon balances [54] and are currently under development [54].

We also did not evaluate the potential effects of climate change or biophysical factors in the scenarios since the model assumed a constant climate for each site, and both the baseline and the selected scenarios would be affected about equally. But considering only the historical baseline, the process model results based on a previous study [6] suggest that increasing atmospheric CO_2_, climate, and N deposition have had relatively significant effects on forest C in our study areas (Additional file 1: Figure S9). The impacts of different climate change scenarios may also influence the effectiveness and feasibility of mitigation activities.

### Policy implications

While various policies mandate the assessment of C stocks on public lands like national forests, currently no national regulations require emissions reductions on public or private lands. Financial incentives like carbon credits, direct subsidies, or tax incentives may be necessary to engage private landowners who may manage forests for profits [41, 50] or to motivate builders and consumers to select wood-based materials [e.g. 37]. Currently, forest landowners can receive carbon credits for avoided conversions, afforestation and reforestation activities, and improved forest management, all of which we found to be effective emissions reductions strategies in the forest ecosystem alone. However, to account for the net effects of mitigation activities, it is important that carbon credit systems account for emissions outside of the forest ecosystem as well as activities that explicitly target emissions associated with HWP pools along with product and energy substitution [8, 33].

The declaration of carbon neutrality for biomass burning is a policy assumption that does not reflect the actual impacts and timing of bioenergy emissions on the atmosphere [54, 58, 54]; [27]. It is often made to encourage the replacement of fossil fuels with bioenergy. Here we evaluate the net impacts on the atmosphere as well as the timing of both emissions and removals, which indicate that in the relatively short-term (up to 32 years in this study), bioenergy use may result in increased carbon emissions.

## Conclusions

This research highlights the importance of taking a systems approach that assesses net emissions from the forest ecosystem, land-use change, HWP, and avoided emissions when evaluating forest sector climate change mitigation scenarios across large, multi-ownership landscapes. The value of applying systems perspectives are increasingly being recognized since emissions reductions in one component (e.g. forest ecosystem) could be offset by an increase in emissions in another component (e.g. product substitution). While some scenarios had considerable mitigation benefits by offsetting emissions from fossil fuel burning or substituting products, these benefits may be negated or outweighed by the increase in C emissions from the products sector or forest ecosystem. Results suggest that implementation of mitigation activities in the forest sector could help reduce the weakening C sink in these study sites.

Based on the scenarios we examined in this 32-year timeframe, maintaining or increasing forestland, extending rotations, and shifting commodities to longer-lived products had the highest mitigation potential. Creating portfolios of multiple land management and HWP scenarios may have considerable mitigation benefits and more realistically reflect the suite of management activities that are often applied across landscapes. We evaluated several bioenergy scenarios and found that bioenergy use may only have a mitigation benefit under longer timeframes and for certain feedstocks such as logging residues which would otherwise go unutilized, but not green roundwood which could instead be used for longer-lived products or remain in the forest to accumulate carbon. Timeframe is also important to consider because the most effective scenario may change over time and some scenarios may require longer timeframes than this analysis period to yield mitigation benefits. Consideration of the displacement of emission-intensive building materials and fossil fuels is critical in ranking mitigation scenarios. However, there is still significant uncertainty in the product and energy displacement, which is likely highly variable between sites and warrants additional research.

Every scenario we examined has tradeoffs, risks, and uncertainties. Designing mitigation scenarios must be locally relevant to the main factors driving C emissions and land management approaches and policies already in place for individual ownerships. The results and conclusions from these two case studies in the U.S. are not universally applicable, but rather are specific to the assumptions and parameters of each mitigation scenario, and to the two regions and timeframe for which they were applied. Carbon management adds another complex dimension to existing forest management objectives. The most effective forest sector mitigation scenarios are likely to be those that achieve atmospheric benefits while also enhancing or retaining co-benefits and ecosystem services such as biodiversity, water quality, and the economy.

## Additional files

**Additional file 1.** Appendix S1. Additional methods and results. 

**Additional file 2.** Appendix S2: CBMF-HWP site-level modeling parameters.

## Abbreviations

BAU: business as usual
C: carbon
CBM-CFS3: Carbon Budget Model for the Canadian Forest Sector
CBMF-HWP: Carbon Budget Modeling Framework for Harvested Wood Products
CH_4_: methane
CO: carbon monoxide
CO_2_: carbon dioxide
CO_2_e: carbon dioxide equivalent
FIA: Forest Inventory and Analysis
GHG: greenhouse gas
Ha: hectare(s)
HWP: harvested wood products
IPCC: Intergovernmental Panel on Climate Change
LLP: longer-lived products
Mg: megagram(s) (one tonne)
N: nitrogen
N_2_O: nitrous oxide
PP: paper products
SC: South Carolina
t: tonne(s)
Tg: teragram(s) (one million tonnes)
TPO: Timber Product Output
UNFCCC: United Nationals Framework Convention on Climate Change
U.S.: United States
WI: Wisconsin
Yr: year
