# Supplementary material for: A systems approach to assess climate change mitigation options in landscapes of the United States forest sector
Source: Carbon Balance Manag. 2018 Sep 4;13:13. doi: 10.1186/s13021-018-0100-x (PMC6123328; doi:10.1186/s13021-018-0100-x)
Supplement: Supplementary file 1 — Additional file 1. Appendix S1. Additional methods and results. [file 13021_2018_100_MOESM1_ESM.docx]

**Additional Materials**

**A Systems Approach to Assess Climate Change Mitigation Options in Landscapes of the United States Forest Sector**

Alexa J. Dugan^*^, Richard Birdsey, Vanessa S. Mascorro, Michael Magnan, Carolyn E. Smyth, Marcela Olguin, Werner A. Kurz

**Corresponding author: Alexa J. Dugan, 610-557-4056,* [*adugan@fs.fed.us*](mailto:adugan@fs.fed.us)

Appendix S1

[**1.0 Additional Methods** 2](#_Toc522189266)

[1.1 Study areas 2](#_Toc522189267)

[1.2 Carbon Budget Model for the Canadian Forest Sector (CBM-CFS3) 2](#_Toc522189268)

[1.3 Input Data 3](#_Toc522189269)

[1.3.1 CBM-CFS3 Parameterization 3](#_Toc522189270)

[1.3.2 Forest Inventory & Growth and Yield Data 4](#_Toc522189271)

[1.3.3 Activity Data 6](#_Toc522189272)

[1.4 Harvested wood products and displacement factors 11](#_Toc522189273)

[1.5 Mitigation Scenarios 14](#_Toc522189274)

[**2.0 Additional Results & Discussion** 18](#_Toc522189275)

[2.1 Model comparison 18](#_Toc522189276)

[2.2 Climate and atmospheric effects 21](#_Toc522189277)

[2.3 Hurricane mitigation scenario results 22](#_Toc522189278)

[**3.0 Supplementary Materials References** 22](#_Toc522189279)

# **1.0 Additional Methods**

## 1.1 Study areas

In consultation with stakeholders, we selected forested landscapes of coastal South Carolina and Northern Wisconsin as the two study sites (Fig. 2) to evaluate historical baseline carbon stocks and climate change mitigation scenarios using the systems based approach. These two particular sites were selected for several reasons. This project was sponsored by Commission for Environmental Cooperation and the three North American governments, so we selected U.S. sites also considering comparable sites in the other countries, while consulting with the study sponsors. Once the sites were selected, we had stakeholder engagement from managers at both sites who helped to formulate mitigation scenarios, considering the most likely mitigation activities that would be deployed in the future.

Also, both sites represent heterogeneous landscapes with multiple ownerships including National Forests private land, and other publicly owned lands, each with different management objectives and approaches. Thus, mitigation scenarios could be tailored and applied to individual ownerships. Also, because each site contains a National Forest—the Francis Marion in SC and the Chequamegon-Nicolett in WI—the historical baseline for all owners could be compared with results from a related study which quantified historical forest carbon stocks and flux on National Forest System lands using several different modeling approaches (Dugan et al. 2017). Privately owned forests in both study areas support the timber industry, thus scenarios that target harvest treatments and the fate of harvested wood commodities could be realistically evaluated.

Furthermore, these two sites have varying forest disturbance regimes resulting in dissimilar age structures, enabling us to evaluate the effects of age structure on carbon stocks. Forests in the coastal South Carolina site were devastated by Hurricane Hugo in 1989, resulting in a current stand age structure dominated by young, productive, regenerating stands. On the other hand, the Northern Wisconsin site has had relatively little disturbance over the past few decades, causing the age structure to be dominated by older, less productive forests (Fig. 3). Lastly, while the Northern Wisconsin site has relatively little urbanization pressure, the coastal South Carolina site contains the city of Charleston which has been rapidly expanding over the past decade and development pressures on forested lands are expected to continue. Overall, these two sites offer distinctive yet representative multi-ownership landscapes with varying disturbance histories and resulting age structures that are suitable for evaluating forest carbon dynamics and the impacts of mitigation strategies.

## 1.2 Carbon Budget Model for the Canadian Forest Sector (CBM-CFS3)

Past, present, and future C stocks for the baseline and mitigation scenarios were modeled using the Carbon Budget Model for the Canadian Forest Sector (CBM-CFS3) (Kurz et al. 2009). We selected the CBM-CFS3 model for analyzing the forest ecosystem and land use change components of our systems approach. Some advantages of CBM-CFS3 are that it is fully consistent with IPCC guidelines for estimation and reporting. It produces results that are fully comparable for different countries, and it is relatively easy to apply compared with some other Life Cycle Assessment models or large-scale Integrated Assessment Models that also represent complex systems (e.g. Running and Gower 1991, Potter 1999, Chen et al. 2000). Other types of models, for example empirical models such as the USFS Forest Vegetation Simulator (Crookston and Dixon 2005), are best used for stand-level analyses, do not consider soil carbon, and are not readily integrated with HWP models. The CBM-CFS3 uses a gain-loss approach which requires a single inventory year plus information on C gain and loss from land use change, disturbances, and growth and mortality. This makes it possible to parse out the effects of disturbance and management on C stocks and emissions. For instance, stock-change models like Carbon Calculation Tool (see Model Validation section) which is readily used in the U.S. for GHG reporting (Smith et al. 2010), uses multiple inventories and interpolates changes in C stocks overtime, thus it is not possible to isolate the effects of individual factors nor conduct mitigation or prospective analyses.

CBM-CFS3 is a growth and yield based ecosystem C model which accounts for C stocks and C stock changes in 10 biomass pools (hardwood and softwood merchantable stem wood, foliage, coarse roots, fine roots, and “other”, which includes branches and seedlings and saplings) and 11 dead organic matter pools (which include woody litter, the soil organic horizon and mineral soil), and emissions of carbon dioxide (CO_2_), methane (CH_4_), carbon monoxide (CO), and N_2_O from slash burning and wildfires. We report emissions from all GHGs together as CO_2_ equivalents (CO_2_e). A Global Warming Potential (GWP) of 25 was applied to methane (CH_4_) (IPCC AR4) and a GWP of 310 was applied to nitrous oxide (N_2_O) (IPCC SAR).

Within the CBM-CFS3 model, carbon enters the forest ecosystem from the atmosphere via photosynthesis. The simulation of disturbances and aging processes causes some carbon to transfer from the biomass to the dead organic matter (DOM) pools. Carbon is transferred among the DOM pools and released back to the atmosphere through processes of decay, decomposition, and disturbances. Carbon can also be transferred to the products sector as a result of harvesting. A temperature-dependent decay rate determines the decomposition for each DOM pool. Stands accumulate carbon following species-specific, age-based growth and yield curves (see section 1.4.1 below). A stand-replacing disturbance resets the stand age to zero and forests then regrow following the same growth trajectory. If a disturbance is not stand-replacing, the stand remains the same age and continues to grow along the same curve’s trajectory (Kurz et al. 2009).

CBM-CFS3 also encompasses land-use change carbon accounting. Afforestation and deforestation are represented as disturbance types in the model with unique transition rules. For instance after afforestation or deforestation events, a change in land-use class to or from forest land-use is triggered. An area afforested or deforested remains in a transitional land-use class (e.g., forest converted to cropland or cropland converted to forest) for 20 years before the land conversion is considered complete and the land-use class changes again to a permanent state (e.g. crop land, forest) (Kurz et al. 2009).

The model simulates past and future (projected) C stocks in the forest ecosystem and is used here to assess the impacts of various land-use change, forest management, and disturbance scenarios. CBM-CFS3 incorporates forest inventory data to determine forest types and stand ages, inventory derived growth and yield curves to model growth rates, and disturbance and land-use change information to ultimately assess the cycling of C through the ecosystem and to quantify C emissions. These key model inputs are described in the next section.

## 1.3 Input Data

### 1.3.1 CBM-CFS3 Parameterization

We employed a spatially-referenced approach consistent with IPCC guidance to simulate forest carbon dynamics with the CBM-CFS3. This means that the model is aspatial in that it represents each stand in a landscape with similar attributes derived from forest inventories, but the actual location of those stands is unknown. To target specific land ownerships and forest types for individual disturbance events and to restrict harvesting to unprotected lands, the inventory datasets were stratified by applying classifiers to each stand. For the coastal South Carolina site: Ownership (private, Forest Service, or other public lands), forest type group (based on the Forest Inventory and Analysis classifications), stand origin (planted or natural), and protection status (wilderness area or unprotected). For the Northern Wisconsin site the same classifiers were applied, except the stand origin classifier was excluded as no mitigation scenarios or disturbance types specifically target planted forests like in the South Carolina site. These classifiers as well as characteristics of the harvests or disturbance activity (e.g. merchantable C removed, minimum age, area impacted) aid to restrict harvests or any disturbance in a given year to specific inventory records. The model then randomly disturbs or harvests eligible inventory records. In section 1.3.3 below (Private and public land disturbance data) we describe the use of Timber Product Output (TPO) to determine the area and merchantable volume harvested by ownership class.

The CBM-CFS3 model contains default parameters such as volume-to-biomass conversions, decay rates of dead organic matter (DOM), DOM turnover rates (Table S1), merchantable tree proportions (Table S2), and temperature based on Canadian administrative units (province or territory) and ecological boundaries (terrestrial ecozones). See appendices 2-5 in Kull et al. 2016 for default parameter values for all provinces and ecozones. Canadian administrative units and ecological boundaries parameters were assigned to the U.S. study sites based on their geographic proximity as well as similar climatic characteristics (e.g. maritime, plains) and terrestrial biomes. For the South Carolina site, the Quebec administrative unit and Atlantic Maritime ecological boundary was assigned. We assigned the Ontario administrative unit and Boreal Shield East boundary to the Northern Wisconsin site. We then edited selected default parameters to better reflect the biophysical conditions in the U.S. sites. For instance, temperature is a critical factor determining decay rate in CBM-CFS3, thus we replaced default temperatures for Canada with 30-year mean annual temperatures for each U.S. study site (PRISM Climate Group). Furthermore, stand initialization carbon stocks for the non-forest soil pool were updated to reflect the average cropland soil C levels in the two study areas—105 t C ha-1 in South Carolina (Potter et al. 2006) and 64 t C ha-1 for Northern Wisconsin (Johnson et al. 2005).

### 1.3.2 Forest Inventory & Growth and Yield Data

Additional information required for simulating carbon dynamics in the CBM-CFS3 were derived from data in the Forest Inventory and Analysis (FIA) database. We selected the 2011 inventory from the FIA database as it corresponds to the most recent year of available forest disturbance and land use change data. However, this inventory year does not coincide with the intended start year of 1990 for the CBM-CFS3 simulation. Thus we followed previously defined steps to roll back the 2011 inventory to the start year of the simulation (Kurz et al. 2016). We first subtracted 21 years from the age of each stand (2011 minus 1990). Any stands with a negative stand age indicate that a stand-replacing disturbance occurred during this roll-back period and require auxiliary information or assumptions to determine the age of the stand prior to the disturbance. Given the extensive forest inventory in South Carolina dating back to 1967, for most stands with an age of < 0 years, we used stand age information from historical inventories to determine stand age prior to the stand replacing disturbance. For the Wisconsin study area, the historical forest inventory data is less extensive, thus a statistical rule-based roll-back algorithm was applied which accounts for stand-replacing disturbances during the historical period to assign ages to any stand with an age of < 0 years. Figure 3 in the main text illustrates the rolled-back 1990 stand ages by forest type for the two study areas.

In the CBM-CFS3 model, growth and regeneration are driven by growth and yield curves which describe the rate of biomass accumulation as a function of stand age. After a stand replacing disturbance such as clear cut harvests, the stand age resets back to zero and the stand regrows following the growth curves trajectory. For non-stand replacing disturbances such as partial harvests or insect outbreaks, the stand age remains unchanged and the stand continues growing along the curves’ trajectory (Kurz et al. 2009).

***Figure S1****. Examples of the growth and yield curves which represent annual carbon accumulation (m^3^ha^-1^) for privately owned lands in a) South Carolina and b) Wisconsin sites. The South Carolina site includes three loblolly/shortleaf pine curves for private ownership: 1) naturally regenerating (orange), 2) plantation (gray), and 3) plantation with 15% increased productivity (yellow) for a mitigation scenario.*


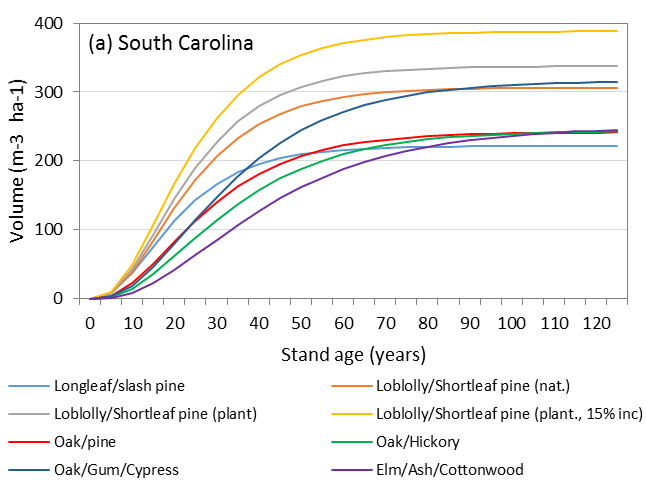

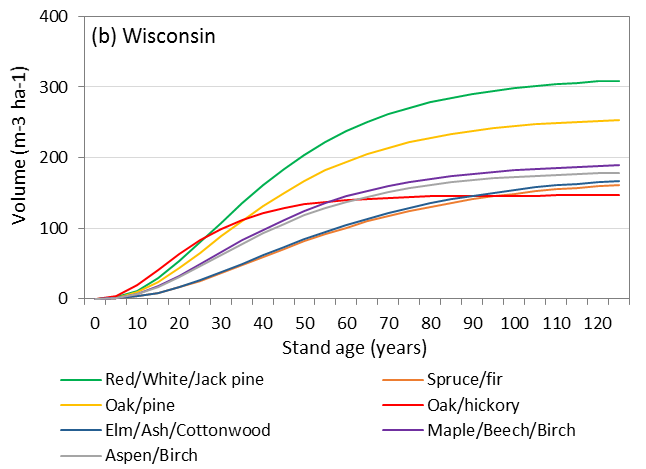


Growth-and-yield curves of projected merchantable volumes indicate the rates of biomass change over time according to stand age, and are used in the modelling framework to simulate regrowth following disturbances. To develop growth curves for each classifier set, we utilized the Carbon On-Line Estimator (COLE) tool (Van Deusen and Heath 2010) which queries the most recent FIA data based on quantitative and qualitative filters to provide growth-and-yield estimates for specified regions. Curves were generated from fully stocked, undisturbed FIA plots for each forest type group and ownership combination within each of the two sites (e.g. Fig. S1). For forest type groups and ownership combinations with fewer than 30 plots, it was necessary to combine ownership groups to achieve a sufficient sample of plots to generate growth curves. For private loblolly/shortleaf pine forests in the South Carolina site, a plantation curve and an increased productivity curve (see mitigation scenarios) were also developed. A total of 13 growth-and-yield curves were generated for South Carolina and 20 for the Wisconsin site.

While these volume curves provide information about the merchantable stem volume, the CBM-CFS3 requires that these volumes as well as all other stand components (e.g., branches, tops, foliage, roots, etc.) be converted to stand-level biomass. The CBM-CFS3 uses a set of equations to calculate merchantable stem volume and convert it to all aboveground and belowground biomass components based on the leading species of each growth-and-yield curve as well as the administrative and ecological boundaries (Kurz et al. 1996; Li et al. 2003; Boudewyn et al. 2007). For each of these multi-species, growth-and-yield curves used for the U.S. sites, the leading species was identified as the most common species found within that forest type group in each study site according the FIA Tree table data (O’Connell et al. 2017). If the leading species was not available in the library of Canadian default species in the CBM-CFS3, a comparable species was selected based on tree architecture. The CBM uses different volume to biomass methodologies and requires stand level conversion factors, thus factors developed from the U.S. inventory (Smith et al. 2003, Heath et al. 2010, Woodall et al. 2011) were not readily transferrable for application in the CBM.

### 1.3.3 Activity Data

The accurate estimation of terrestrial carbon dynamics requires information on forest disturbances characterized by type of disturbance, ideally on an annual basis to capture the variability of disturbance rates (Spalding 2009; Kurz 2010). To improve our understanding of their particular impact over the carbon pools, we need to know what happened, when it happened, and how each disturbance type affected the distribution of the carbon stored in the vegetation and soils, and subsequent emissions into the atmosphere (Mascorro et al., 2014). Remote sensing data offers a cost-efficient way to monitor disturbances large areas on a regular basis, including areas that are otherwise difficult to access (Coops et al. 2006; Wulder & Coops, 2013).

#### Land-use change

Land use changes including deforestation, afforestation and reforestation are important drivers of forest C change. Given the lack of specific land-use change products for these two study areas, we used land cover change during the historical period (1991-2011) from the National Land Cover Database (NLCD) as a proxy for land-use change. This data was derived from Landsat satellite imagery at a 30m spatial resolution for the conterminous of U.S. (Fry et al. 2011) labeling land cover change areas using a set of knowledge-based rules and a decision tree algorithm (Homer et al., 2015).

For the period from 1991 to 2000, we applied the average annual area deforested and afforested from the NLCD 1992/2001 retrofit product which utilizes decision tree classification of Landsat imagery from 1992 and 2001 to determine land cover change at the Anderson Level 1 classification scale (Fry et al. 2009). The Anderson Level 1 classification scale includes both forested and non-forested wetlands, thus we excluded cover changes between wetlands and forest from the land-use change analysis and only included changes to or from forests as land use changes.

For the period from 2001 to 2011, the NLCD 2001 product was compared to the NLCD 2011 product to calculate the average land use change for the three ownerships (Table S1a-b). This classification is based on a 16-class, Anderson Level II land cover classification scheme. Any pixels which were forest in 2001 and non-forest in 2011 were coded as deforested and those that were non-forest in 2001 and forest in 2011 were coded as afforested.

While the NLCD includes some clear land use changes such as forest to settlement, there are limitations of using a land cover change product to approximate land-use change. For instance, the use of NLCD as a proxy for land-use change may cause an overestimation of deforestation and reforestation particularly in areas that experienced clear-cut harvest. More specifically a harvested area may be classified as a cover change from forest to grassland, but the land use does not change. However, this would only impact the baseline scenario results, not the evaluation of mitigation scenarios. This is because the levels of land-use change and disturbance were applied equally in the baseline and each mitigation scenario (unless they were the targeted mitigation activity) essentially factoring each other out in order to isolate the impacts of the mitigation action. Even for mitigation scenarios targeting land use change or harvest rates, we generally targeted a percentage increase or decrease in the activity relative to the baseline and did not focus on the total area impacted, thus a potential overestimation of land-use change would not have a significant impact in the evaluation scenarios.

***Table S1.*** *Total area of land use change (hectares) from 1991-2000 and 2001-2011 for a) coastal South Carolina and b) Northern Wisconsin. Data was compiled from the National Land Cover Change datasets.*

1. South Carolina

|  | Deforestation | Afforestation | Net |
| --- | --- | --- | --- |
| *Forest Service* | | | |
| 1991-2000 | 1,078 | 1,601 | *Gain* |
| 2001-2011^a^ | 186 | 600 | *Gain* |
| *Public* | | | |
| 1991-2000 | 965 | 1,117 | *Gain* |
| 2001-2011^a^ | 408 | 155 | *Loss* |
| *Private* | | | |
| 1991-2000 | 23,812 | 9,790 | *Loss* |
| 2001-2011^a^ | 14,342 | 4,748 | *Loss* |

1. Wisconsin

|  | Deforestation | Afforestation | Net |
| --- | --- | --- | --- |
| *Forest Service* | | | |
| 1991-2000 | 983 | 1,558 | *Gain* |
| 2001-2011^a^ | 4,230 | 541 | *Loss* |
| *Public* | | | |
| 1991-2000 | 4,283 | 5,982 | *Gain* |
| 2001-2011^a^ | 19,683 | 2,268 | *Loss* |
| *Private* | | | |
| 1991-2000 | 22,749 | 11,004 | *Loss* |
| 2001-2011^a^ | 20,977 | 5,011 | *Loss* |

*^a^ Average annual afforestation and deforestation rates applied to the period 2012-2050.*

The average annual afforestation and deforestation rates from 2001 to 2011 were applied to the rest of the simulation (2012-2050) for the baseline scenario. Given limited data on the disposition of deforested wood, we assumed that wood was left on site rather than transferred to product pools. We did not conduct afforestation events during the historical period as the 1990 inventory would have already contained all land afforested from 1990-2011 and there is little evidence to determine which records were afforested versus representing regrowth from disturbances.

#### National Forest disturbance data


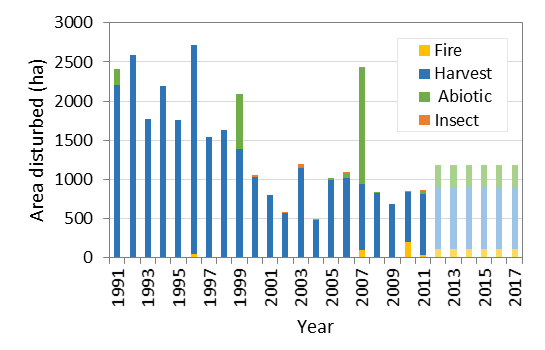


***Figure S2****. Area disturbed by type from 1991-2017 for the Chequamegon-Nicolet National Forest within the Northern Wisconsin study site. Lighter bars represent 10 year average disturbance rates.*

For the National Forest lands within the two study sites, we utilized spatially-explicit, manually-verified disturbance maps including type and magnitude which were generated for all National Forests in the U.S. as part of a related study (Healey et al. 2014, Healey et al. 2018). These maps were generated first by running the Vegetation Change Tracker (VCT) algorithm (Huang et al. 2010) on Landsat maps during this period to detect annual forest change. A process of manual verification and disturbance type attribution using resources such as the Monitoring Trends in Burn Severity (MTBS) database, a spatial database of historical harvesting activities (National Forest System Forest Service Activity Tracking System—FACTS) and insect disturbance records (Johnson and Wittwer 2008).

Disturbances include harvests, fires, insects, and abiotic (windstorms, hurricanes) (e.g., Fig. S2) and intensity based on the percentage change in canopy cover for the period 1990-2011. We consolidated the intensities into two classes: stand replacing (>50% change in canopy cover) and non-stand replacing (< 50% change in canopy). For fires from 1991-2011, 23% were stand replacing in the South Carolina site and 28% in the Wisconsin site. We then overlaid these disturbance maps on a forest type group map (Ruefenacht et al. 2008) to extract the area disturbed by forest type and disturbance type and magnitude. We applied the average annual area disturbed by type and magnitude for each ownership from 2012-2050 for the baseline scenario. Given the uncertainty and randomness of fires, we did not target specific forest types after 2011. Insects were excluded from the period of the mitigation scenarios (2018-2050) as they had a minor impact and were not specifically targeted in the mitigation scenarios.

#### Private and public land disturbance data

***Commercial harvests***

Harvesting is the dominant disturbance affecting both study sites. To determine the volume of wood harvested annually during the historical period we utilized data compiled in the Timber Product Output (TPO) database which reports roundwood removals and logging residues for each county by variables such as ownership, type of wood (hardwood, softwood), and timber products, roughly every 2-5 years depending on state since the mid-1990s. For the South Carolina site, TPO data is available every 2 years from 1995-2011. We applied the annual roundwood removals by ownership type and wood type from 1995 for the period 1991-1995, the removals from 1997 for 1996-1997, the removals from 1999 for 1998-1999, and so on. For public lands in SC, removals data only extends to 2003, thus we applied the annual volume of roundwood removals from 2003 through 2011. For the Wisconsin site, TPO data is available every 5 years from 1996 to 2011. Thus we applied the annual removals by ownerships type and wood type reported for 1996 for the period 1991-1996, the removals from 2001 for the period 1997-2001, and so on.

The removals were also divided into the proportion of partial cut versus clear cut based on data from Oswalt and Smith (2014). In the U.S., partial cutting is more prevalent than clearcutting, however, clearcutting is still common, especially in managed plantations in the Southern U.S. (Oswalt and Smith 2014). In the South Carolina site, partial harvests were assumed to remove 50% of the merchantable trees, while clearcut harvests remove 95% of the merchantable trees. For the Wisconsin site, we assumed that partial harvests remove 25% of the merchantable trees (Peckham et al. 2013) and clearcuts remove 85%. We assumed that 29% of the residues in Wisconsin and 40% of the residues in South Carolina were collected and transferred to the product sector after clearcut harvests according to TPO data (e.g. Haugen 2013).

For the South Carolina site, the minimum harvest ages are 23 years old for softwood and mixed stands and 38 years old for hardwood stands. In the Northern Wisconsin site, minimum harvest ages are 20 years old for Aspen-birch, 40 years old for Red/White/Jack pine, Spruce-fir, and Maple-Beech, 60 years old for Oak-Hickory and Elm/Ash/Cottonwood forest types (Mills and Kincaid 1992).

The CBM-CFS3 requires that the harvested wood targets be in merchantable C units (tonnes). Therefore the volume of roundwood removals were first converted to biomass using specific gravities from the FIA database (USFS 2015) and equations for calculating biomass from Woodall et al. (2011). Biomass was converted to C by multiplying biomass by 0.5 based on the assumption that biomass is 50 percent carbon (Birdsey 1992).

***Fires, Insects, Abiotic disturbances***

To derive annual estimates of area disturbed by disturbance type on public and private lands, we compiled multiple disturbance datasets available for the two study areas from different agencies. Annual forest disturbance maps were retrieved from 1991 to 2010 from the North American Forest Dynamics (NAFD) and clipped to the study area of Wisconsin. The NAFD product provides annual maps of land cover changes without attribution derived from Landsat at 30m resolution from 1986 to 2010 (Huang et al. 2010, Healey et al. 2005, Goward et al. 2015).The NAFD produces a map where each pixel is labeled as a persistent class (i.e. forest, non-forest, water), or as disturbed.

Areas of disturbance in South Carolina were retrieved annually from 1991 to 2011 from the Landscape Change Monitoring System (LCMS). The LCMS is a novel framework that integrates Landsat remote sensing products and ancillary datasets to characterize the year, type, severity, and disturbance cause and has been tested in various pilot study areas across the U.S., but is not yet available nation-wide (Schroeder et al. 2017).

Characterizing the disturbance type on the landscape is crucial to know their corresponding effect on the redistribution of carbon in the terrestrial carbon pool and subsequent atmospheric emissions (Kurz 2010; Turner 2010). Following the Mascorro et al. (2014) approach and using ancillary data sources (described below) we attributed the forest disturbances detected by the LCMS and NAFD to fires, insects, or abiotic factors and then generated annual rates of disturbance by type from these maps (Fig. S3).

To attribute disturbed areas to fire, data on fire disturbances were retrieved for both sites, Wisconsin and South Carolina, from the Land and Atmosphere Near real-time Capability for EOS (LANCE)/ Fire Information for Resource Management System (FIRMS) database (ESDIS 2015) for the years 2001-2010 and 2001-2011 respectively. The FIRMS data is derived from MODIS satellite imagery and is comprised by fire points of hotspot locations depicting the center of a 1km pixel. In both sites, we filtered the data and selected only the hotspots with a confidence level higher than 90%. A buffer of 500m was then created around each point to depict the area encompassed by a 1km pixel.

Additional fire data was available from the Monitoring Trends in Burn Severity (MTBS) database (Eidenshink et al. 2007). The MTBS project maps burn severity and fire perimeters for fires greater than 500 acres in the east and 1000 acres in the west - from 1984 onwards. These polygons were combined with the FIRMS hotspots to create a single unified annual map of areas disturbed by fires.

For the Wisconsin site additional fire occurrence data were also retrieved from the Department of Natural Resources (DNR) from 1991 to 2011. For the coastal South Carolina site, we supplemented the spatially-explicit fire data with county-based data of the annual area burned from the South Carolina Forestry Commission (SCFC). We first subtracted the annual area burned derived from the National Forest disturbance dataset and the area burned attributed from the ancillary data (FIRMS, MTBS) from the SCFC area burned data and assumed that the remaining area burned occurred on public or private lands. We assigned additional area burned to public and private lands based on the relative proportion of forested area in each ownership class.

To attribute disturbed areas to insect or abiotic factors (Fig. S3), we utilized the U.S. Forest Service National Insect and Disease Survey (IDS) data which provides a consistent source of aerially detected insect, disease, and abiotic forest damage from 1997-2010 on forested lands across the U.S. as part of the Forest Health Monitoring and Forest Health Protection program (Johnson and Wittwer 2008). We distinguished defoliation insect disturbances from other insect disturbances causing mortality or dieback. However, we excluded all low-severity defoliation events (< 50% defoliation) given their low impact on C dynamics and the low likelihood that these often seasonal events were captured by the NAFD and LCMS disturbance products.

| a)  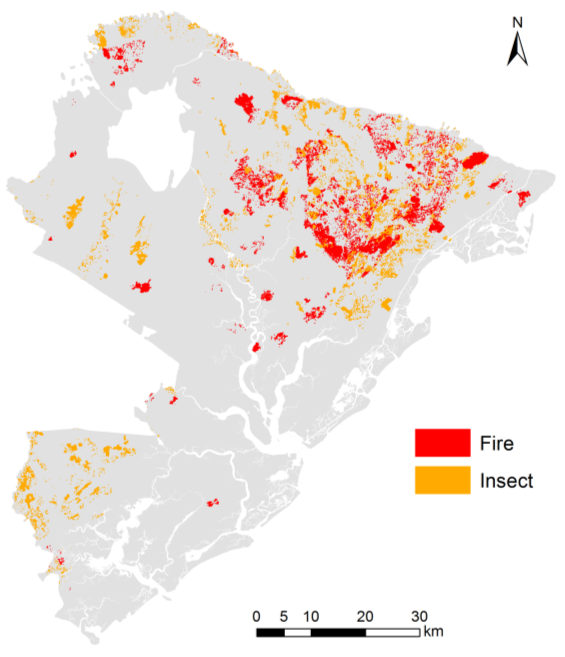 | b)  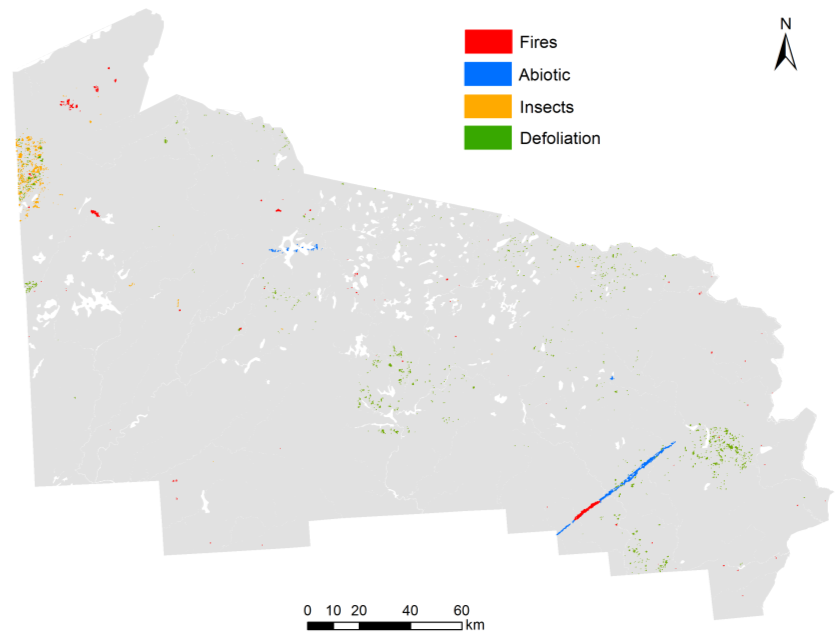 |
| --- | --- |

*Figure S3. Forest disturbances attributed by type from the LCMS data source from 1991 to 2011 in a) South Carolina, and from the NAFD datasource from 1991 to 2010 in b) Wisconsin.*

## 1.4 Harvested wood products and displacement factors

Carbon harvested from the ecosystem (roundwood, salvage, and residues) was then transferred to the commodity stream. To estimate HWP emissions, we applied the Carbon Budget Modelling Framework for Harvested Wood Products (CBM-FHWP) which tracks the harvested C and emissions through the lifecycle of manufactured commodities, burning of fuelwood (bioenergy), mill residue use and waste, and exported products, and to retirement in landfills (Fig. S4). The CBM-FHWP model does not account for emissions from transportation, preprocessing, or drying. It also does not account for dimensions of the wood, but rather splits the roundwood into commodity classes which each have associated in-use half-lives based on their size and uses. Durable wood products such a wood used to build houses and furniture have much longer half-lives than pulp and paper products. Fuelwood products (bioenergy) are assumed to release all carbon the year they were produced (i.e., instantaneous oxidation).

Data on the fate of harvested wood were obtained from both regional and national statistics. TPO data summarized in state timber industry assessments every 2-5 years (e.g. Johnson and Smith 2007; Haugen 2013) provide information on the quantity of harvested roundwood manufactured for seven commodities classes including saw logs, veneer logs, pulpwood, composite panels, fuelwood (bioenergy), posts/poles/pilings, and other industrial products (Fig. S5), as well as the disposition of mill residues to commodities, bioenergy, or disposal. The model accounts for processing losses in the form of mill residues that may be used (pulpwood or bioenergy) or unused (landfilled). In both sites, roughly half of mill residues were used for bioenergy, while the other half was used in solid wood commodities, and less than 1% were landfilled (Appendix 2). We assumed that all harvested roundwood was used locally, but we applied export statistics to the processed commodities. National statistics on the proportion of commodities exported outside of the U.S. versus consumed domestically were obtained from the U.S. Timber Production, Trade, Consumption, and Price Statistics, 1965–2013 Report (Howard and Jones 2016, Table 5a).


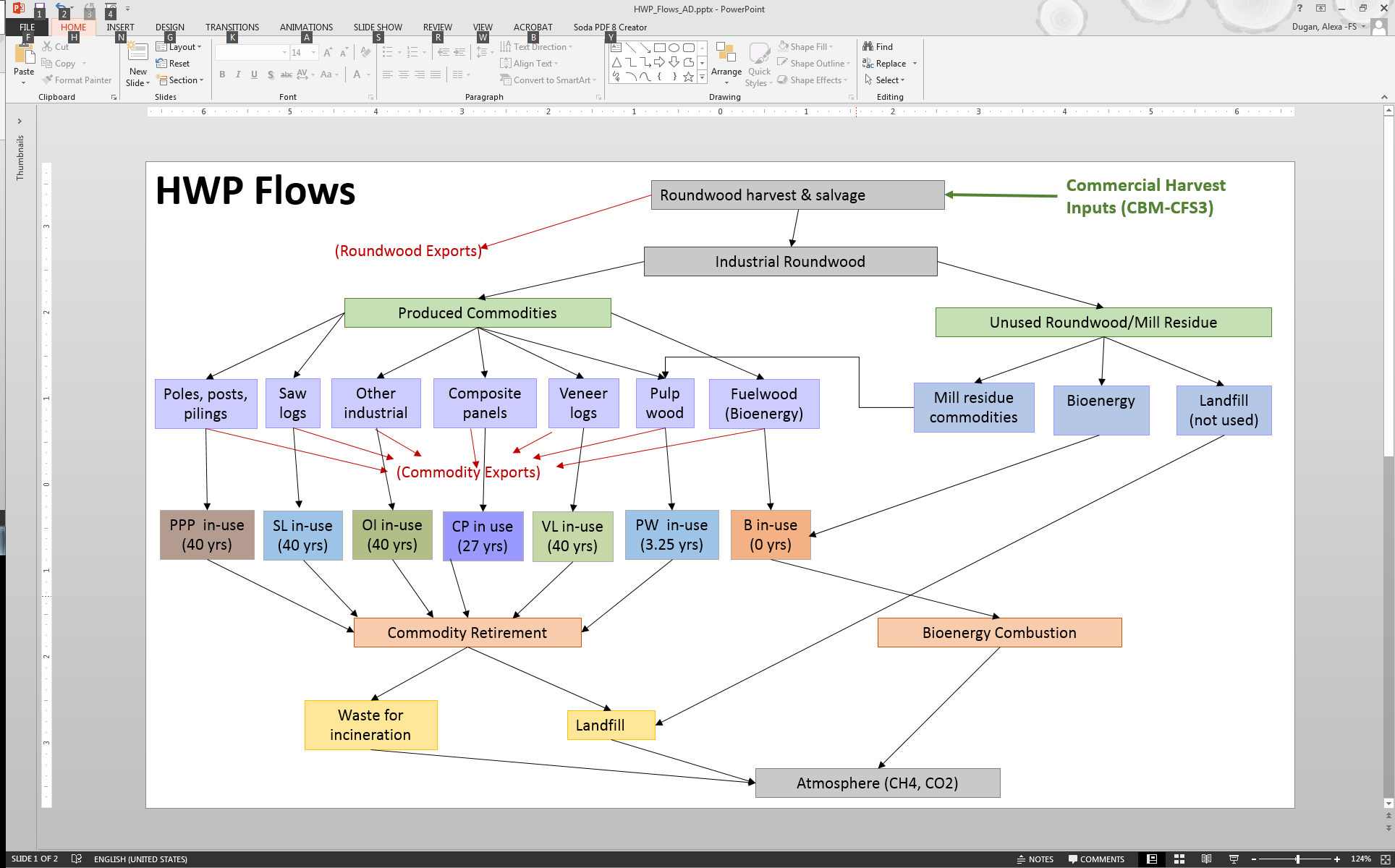
***Figure S4.*** *The fate of carbon in harvested wood products (HWP) stocks and emissions through the lifecycle of manufactured commodities, exports, milling, retirement, and bioenergy combustion. The parameters applied to each stage in this cycle are provided in the attached spreadsheet (Appendix 2).*

Commodity in-use half-lives were assumed to be 40 years for saw logs, other industrial, and posts, poles, and pilings and 30 years for veneer logs, based on average values from Skog (2008) and IPCC (2013), 27 years for composite panels, 3.25 years for pulpwood (IPCC 2013), and zero years for fuelwood (bioenergy) assuming instantaneous oxidation. At the end of its in-use period, we assumed that all wood was retired in municipal solid waste (MSW) or industrial solid waste (IWW) landfills where products decay based on decay rate constants derived from Canadian territories and half-lives from averages of IPCC default values for dry and wet, as well as temperate climates (IPCC 2006). We did not consider recycling of products or cascaded wood use. We did not consider emissions of waste wood with energy capture because according to a 2015 report by the U.S. Energy Information Administration there are only 71 waste-to-energy plants in the U.S., most of which are concentrated in the northeast and none of which are located in South Carolina or Wisconsin (<https://www.eia.gov/todayinenergy/detail.php?id=25732>). For all harvested wood products parameters, 10-year averages of the historical values were applied to the simulation period from 2012 through 2050. See the attached Appendix 2 for detailed information on HWP modeling parameters used for each site.


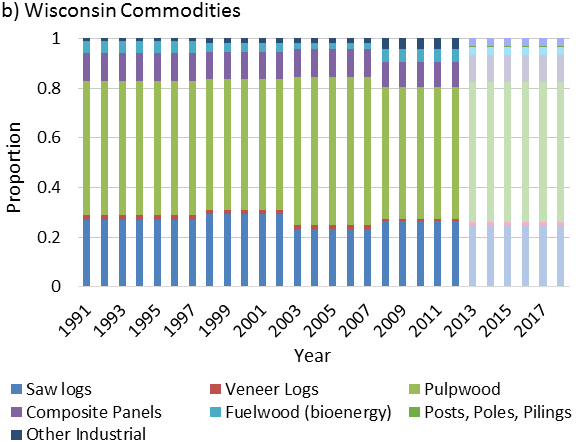

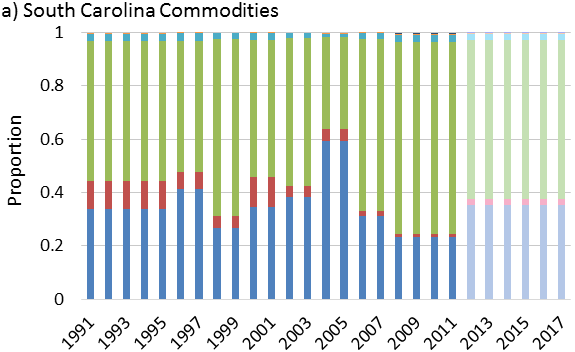


***Figure S5.*** *Annualized proportions of roundwood removals in each commodity class for the a) South Carolina site and b) Wisconsin site, derived from the FIA Timber Product Output reports. Lighter colored bars from 2012-2017 indicate 10 year averages applied from 2012 through 2050.*

This systems approach also considered displaced emissions defined as the emissions that would have been released if alternate fossil fuel sources (e.g. coal, natural gas, oil) or more fossil fuel intensive building materials had been used instead. Product displacement depends on the amount of energy needed to extract, transport, manufacture assemble and operate wood materials as opposed to alternative materials such as steel, concrete or plastic. Displacement factors are used to describe the amount of C displaced per amount of carbon used in the wood product (Sathre and O’Connor 2010). We utilized the following average displacement factors, calculated at the national level in Canada: 0.54tC displaced per tC of sawnwood (saw logs and veneer logs), 0.45 tC displaced per tC of panels (composite panels), and 0.89 tC displaced per tC of bioenergy (Smyth et al. 2017).

Displacement factors for wood substitution were based on the relative emissions from a more wood-intensive product versus a less wood intensive product for potential end-uses including single family home, multi-family home, multiuse building, furniture, flooring, and decking. The wood product displacement was further broken down by sawnwood and panels as these are the main long-lived wood commodities contained in end use products. Sathre & O’Connor (2010) found that product displacement factors ranged from -2.3 to 15.0 tC/tC with an average value of 2.1. The extreme variability in these estimates is because the meta-analysis included studies using a wide range of end-use products which differed from those used in our analysis, as well as systems boundaries which varied by study (may or may not include forest ecosystem emissions, operational emissions, post-consumer emissions from landfills, or bioenergy and substitution of fossil fuels from postconsumer products). As a result, several studies have called into question the application of the Sathre & O’Connor average product displacement value (Smyth et al. 2017, Suter et al. 2017, Chen et al. 2018).

Displacement factors for bioenergy substitution were derived from comparing bioenergy facility emission-intensities to those for the extraction, transportation and conversion of fossil fuels for heat or electricity including coal, fuel oil and diesel, and natural gas. Typically bioenergy displacement factors range from less than 0.5 to up to 1.0 (Sathre and O’Connor 2010). Smyth et al. 2017 produced two average displacement factors for bioenergy based on different bioenergy feedstock supplies. The first, 0.89 tC/tC, assumes a *constrained* supply of bioenergy is produced to match the demand of fossil-fuel based heat. The second, 0.47 tC/tC, assumes that all collected residues are converted to bioenergy, and when local energy demands are met excess biomass is converted to electricity causing the displacement factor to decline. We selected the higher bioenergy displacement factor of 0.89 tC/tC to assess a high potential displacement benefit of bioenergy. See Smyth et al. 2017 for a complete description of the displacement factor estimates. To calculate the avoided emissions for each product type, the displacement factor is then multiplied by the quantity of C utilized for each end-use product.

Given the limitations of directly applying the Sathre & O’Connors factors for product substitution, and the lack of published displacement factors developed specifically for the U.S. and for the system boundaries used in this study, we accepted the Smyth et al. 2017 displacement factors derived for Canada as the best available option.

## 1.5 Mitigation Scenarios

The broad goal of climate change mitigation in the forest sector is to remove additional carbon dioxide from the atmosphere or reduce emissions by (1) increasing the rate of net carbon uptake by forests, (2) increasing carbon stocks in forests and harvested wood products, (3) substituting wood biofuel for fossil fuels, and (4) substituting wood products for other materials that have high fossil carbon emissions during production. In the context of U.S. climate mitigation policies and programs, these activities represent practical ways to sustainably manage forests for climate mitigation while also minimizing tradeoffs with other ecosystem services and if possible, increasing production of forest commodities.

A baseline “business as usual” (BAU) scenario involves the projection of current trends based on the average of recent past observations of disturbance and management, deforestation and afforestation events over the last 10 years of the historical period (2002-2011). For each scenario the baseline rates of disturbance are applied from 2012 through 2050 unless a particular activity is otherwise targeted by the mitigation activity. Insect disturbances were excluded from the simulation period due to low impact and high uncertainty in the affected area. For fires and abiotic disturbances specific forest types were not targeted given high uncertainty in forest types affected. All mitigation scenarios begin in 2018. We selected and evaluated eight mitigation scenarios for the coastal South Carolina site, including two that relate to a hurricane (Table 3, main text) and six for the Northern Wisconsin site (Table 4, main text) based on their potential for occurrence and relevance to the region. For each region, the mitigation scenario is evaluated against the baseline scenario. We selected these scenarios after consultation with stakeholders in each of the regions.

***South Carolina Mitigation Scenarios***

**Scenario 1 – Increase residues:** This scenario increases the use of wood for bioenergy and reduces emissions by more efficiently utilizing logging residues. To maintain productivity and ecological functions, harvest guidelines generally indicate that at least 30% of the logging residues are generally retained on the site to maintain productivity (Walmsley and Godbold 2010; Trottier-Picard et al. 2014; Adamczyk et al. 2015). We simulate removal of 70% of un-utilized logging residues from the forest which represents the maximum residue utilization rate possible. These additional residues then enter the harvested wood consumption stream for bioenergy. Results will reflect the increased utilization compared with the baseline levels of 40% utilization (Oswalt et al. 2014). The accounting includes a full analysis of the substitution of bioenergy for the alternative fossil fuel source using a “displacement factor” approach.

**Scenario 2 – Increase productivity:** This scenario increases productivity of managed forests by 15% through advanced silviculture, genetics, and site management. We do not propose specific silvicultural or other land management practices, but rather, assume that land managers may apply a range of practices that result in an average increase in productivity of 15%. The scenario targets 50% of existing and/or afforested loblolly pine plantations (private lands) per year which are ≤ 12 years old. To simulate this increased productivity, stands affected follow an a new growth curve generated by increasing the modeled loblolly pine planation growth curves by 15% for the duration of the simulation period, and comparing the results with the baseline BAU scenario.

**Scenario 3 - Reduce deforestation:** A pair of mitigation scenarios is aimed at reducing forest loss on privately owned lands in two different ways. The first reduces the area deforested per year by 25% of the total annual area deforested from the baseline scenario. The baseline rates of afforestation and deforestation results in net loss of approximately 857 ha annually during the simulation period. In this scenario, the deforestation rate on private lands is reduced from 1304 ha per year from the baseline scenario to 978 ha per year resulting in a net loss of approximately 532 ha annually across all three ownership classes (Fig. S6).

**Scenario 4 – No net loss:** This mitigation scenario is also concerned with reducing the area of forest loss on privately owned lands by offsetting the area deforested with afforestation or reforestation. This no net loss scenario maintains the baseline deforestation rates, but increases the afforestation rate so that the area afforested annually is equal to the area deforested annually. This scenario causes a 3-fold increase in the afforestation rate on private lands from 432 ha per year to 1304 ha per year. Because afforestation and deforestation rates on public and national forest lands are unchanged, this scenario results in a small net gain of approximately 15 ha per year (Fig. S6).

**Scenario 5 – Longer lived wood products:** To target the solid wood products sector, we increased long-lived wood products by a total of 10% per year divided equally among saw logs, veneer logs, and posts/poles/pilings, while decreasing paper products (pulpwood) by 10% per year. Total removals are not changed, only the product mix is altered as simulated by the harvested wood product model.

**Scenario 6 –Bioenergy:** The proportion of harvested wood used for bioenergy (fuelwood) production is increased by 10% by reducing an equivalent amount of long lived wood products.

**Scenario 7 – Hurricane Hugo Salvage:** Another pair of scenarios addresses the potential impacts of a future hurricane similar to the devastating 1989 Hurricane Hugo. Hurricane Hugo was a category 4 storm that came ashore just north of Charleston, SC, damaging more than 4.5 million acres of timberland. Of the softwood inventory in Berkeley and Charleston counties, 48% was killed and 53% damaged by Hurricane Hugo. Hardwood mortality totaled 16% while 57% of hardwoods were damaged in the hurricane The hurricane affected 79% of national forest or other public lands and 68% of private lands. Publically managed lands contained a higher proportion of older and larger trees which are more susceptible to wind damage. The historical salvage rates after Hurricane Hugo were 7.6% of growing stock hardwoods (1.2% of mortality) and 26% of growing stock softwoods (13% of mortality) (Sheffield and Thompson 1992). To achieve a stand age structure similar to the age structure after Hurricane Hugo, the stand age was reset for 70% of the stands affected by the Hurricane. Comparing historical post-Hugo harvest rates in the South for stands affected versus not affected by the hurricane showed a significant reduction in harvesting after the hurricane. Thus the rate of harvesting was reduced by 60% to approximately 1991-1995 levels for a decade after the hurricane. Thereafter, harvesting rates were gradually increased by 10% every two years until they reached pre-hurricane levels. The emissions of the hurricane with Hugo salvage rates were evaluated against a “no salvage” baseline Hurricane scenario with no salvage logging.


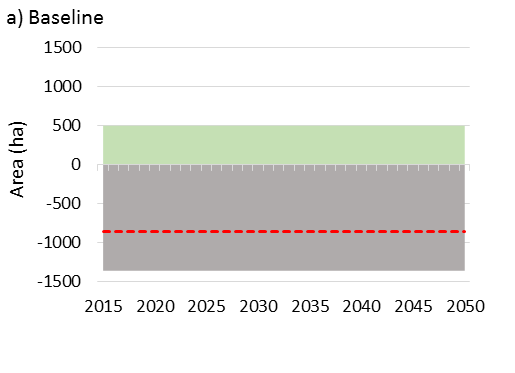

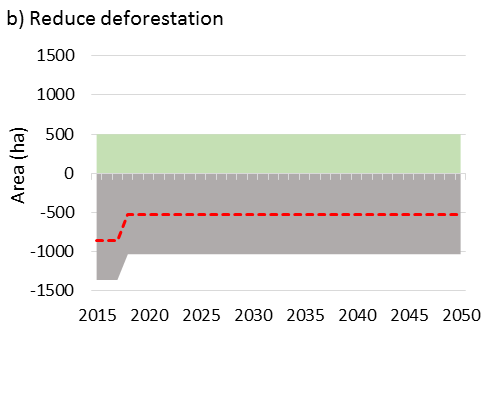

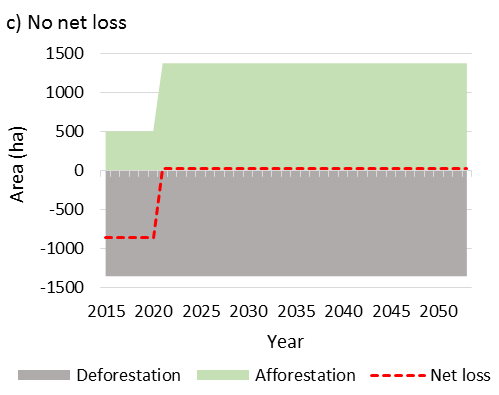


***Figure S6****. Comparison of the afforestation and deforestation rates for the South Carolina study site for the a) baseline, b) reduce deforestation, and c) no net loss scenarios from 2015-2050.*

**Scenario 8 – Hurricane + increase salvage:** For this second hurricane scenario, the same hurricane is simulated in 2018, but the salvage rates are increased to 30% of growing stock hardwoods (14% of mortality) and 60% of growing stock softwoods (31% of mortality). The mitigation effect of increased salvage logging is evaluated against the baseline Hurricane scenario (no salvage) and the Hurricane Hugo salvage scenario (scenario 7).

***Wisconsin Mitigation Scenarios***

**Scenario 1 – Increase residues:** This scenario increases the use of wood for bioenergy and reduces emissions by more efficiently utilizing logging residues. To maintain productivity and ecological functions, harvest guidelines generally indicate that at least 30% of the logging residues should be retained on the site to maintain soil nutrients and productivity (Walmsley and Godbold 2010; Trottier-Picard et al. 2014; Adamczyk et al. 2015). We simulate removal of 70% of un-utilized logging residues from the forest which represents the maximum residue utilization rate possible. These additional residues then enter the harvested wood consumption stream for bioenergy. Results reflect the increased utilization compared with the baseline levels of 29% utilization (Haugen 2013). The accounting includes a full analysis of the substitution of bioenergy for the alternative fossil fuel source using a “displacement factor” approach.

**Scenario 2 – Increase harvests:** The goal of this mitigation scenario is to increase harvests of forests explicitly for biomass energy as a substitute for fossil fuels. Since forest growth exceeds mortality plus removals for wood products, it may be possible to increase the harvest of forests specifically for bioenergy production without reducing growing stocks, though the rate of accumulation would decline. In this scenario we reduce by half the gap between net growth and removals by increasing the merchantable C harvested (public and private land) and area harvested (national forest land) by 10% from baseline levels. All of the wood from the increased harvests are utilized for bioenergy production. To achieve this, additional clear cut and partial cut harvest disturbance types were generated in CBM to distinguish increased harvest used specifically for bioenergy from the baseline harvests used for the assortment of products. We analyze impacts on ecosystem C stocks and substitution of bioenergy for the alternative fossil fuel source using a “displacement factor” approach.

**Scenario 3 – Extend rotation:** This mitigation scenario seeks to increase carbon stocks in the ecosystem and harvested wood products by extending the rotation length and increasing the proportion of long-lived wood products. In this scenario we increase average rotation length by reducing the merchantable C harvested (public and private land) and area harvested (national forest land) by 10% from baseline levels. We also achieve a reduction in rotation length by increasing the minimum harvest age by 10 years for all forest types. The proportion of harvests used for long-lived wood products is increased by 5% at the cost of paper products.

**Scenario 4 – Increase LLP:** Long-lived wood products (saw logs) are increased by a total of 10% per year, while paper products (pulpwood) are decreased by 10% per year. Total removals are not changed, only the product mix is altered as simulated by the harvested wood product model.

**Scenario 5 – Increase bioenergy:** The proportion of harvested wood used for bioenergy production is increased by 10% by reducing an equivalent amount of long lived wood products. For scenario 4 and 5, total removals are not be changed, only the product mix is altered as simulated by the harvested wood product model.

# **2.0 Additional Results & Discussion**

## 2.1 Model comparison

The CBM-CFS3 model was originally developed for carbon accounting in Canada but has since been parameterized for other countries (e.g., Pilli et al. 2013, Olgiun et al. 2018). While we parameterized the CBM with U.S. values including growth and yield models, disturbance types and matrices, climate, and soil carbon initialization values, and utilized U.S. forest inventory and disturbance data, some CBM default parameters such as biomass conversion factors were selected to match as closely as possible the U.S. forest conditions.

To validate the use of the Canadian model for U.S. sites, we compared historical baseline results (1990-2016) for the forest ecosystem from the CBM-CFS3 model with ecosystem carbon estimates derived from forest inventory data in the U.S. The Carbon Calculation Tool (CCT) is an empirical based model which summarizes the available forest inventory data and has been used to meet National Greenhouse Gas Inventory reporting requirements in the U.S. CCT estimates total C stocks and C stock change multiple survey years using allometric equations and tree-level measurements (Smith et al. 2010; Woodall et al. 2011). For National Forest lands within each study site, we also validated CBM-CFS3 results against results of a process model, the Integrated Terrestrial Ecosystem Carbon (InTEC) model, which was run across each National Forest in the U.S. as part of a related study (Dugan et al. 2017).

Results of the model comparisons for the period 1990-2016 for each study site and ownership type indicate that the CBM-CFS3 compared reasonably well to the FIA data as summarized by the CCT model (Figs. S7-S8). For the South Carolina site (Fig. S7), the C stock estimates from CBM are roughly 4-24% higher than the CCT estimates, though C stock estimates are always closer in more recent years. For the Wisconsin site (Fig. S8), the C stocks estimated by the CBM model are roughly 4-10% higher than the CCT, depending on the year and ownership. C stocks estimated by InTEC are 1-10% higher than CBM estimates for South Carolina and only 1-2% higher than CBM results for the Wisconsin site. The results of CBM and InTEC model for the Forest Service lands are very similar especially for the Chequamegon-Nicolet National Forest in the Wisconsin site. The InTEC results for Forest Service lands in South Carolina also contain some areas outside of the study area (Sumter National Forest) which likely explains the small discrepancies.

The small discrepancies in forest C density are due in part to differences in forested areas. For instance, in CCT the forest area changes annually due to land use changes tracked over survey years. Changes to the inventory design and protocols in the early 2000s have caused some known discontinuity in the FIA forested area and also the C stock estimates from CCT (Woodall et al. 2011; Goeking 2015). This is most evident in the CCT results for South Carolina which show a sharp increase in the late-1990s and early 2000s. For the CBM model, a single inventory year is used, in this case, 2011. The forest areas from the 2011 inventory are then rolled back to the start year (1990) based on land-use change rates, while stand ages are rolled-back based on ancillary data and a rule-based algorithm (previously described). On the other hand, the InTEC model uses a single forest type map over the modeling period thus forest area does not change.

***Figure S7.*** *Comparison of total C stocks (Mg C ha-1) modeled using the Carbon Budget Model versus the Carbon Calculation Tool (Smith et al. 2010) and Integrated Terrestrial Ecosystem Carbon model (Dugan et al. 2017) for (a) all ownerships, (b) public, (c) Forest Service, and (d) private lands in the coastal South Carolina study site. Results from the InTEC model also contain South Carolina’s Sumter National Forest.*

Furthermore, the models use different datasets. Both the InTEC and CBM models integrate high-resolution Landsat-derived disturbances whereas CCT does not explicitly track disturbances, but rather detects their effects on forest growth rates, age structure, and mortality measured during the inventories. Because individual plots are only surveyed every 5-10 years, the effects of more recent disturbance may be lacking from the CCT estimates.

Lastly the models use very different methodologies and parameters. Although each model is closely tied to the forest inventory, CBM estimates C stocks and flux using a gain-loss method, while the CCT employs a stock-change method. The gain-loss method calculates C stocks in an initial inventory year and then estimates inter-annual changes in C stocks based on net increment (growth minus mortality) as well as losses due to disturbances, decomposition, and land use change. Conversely the stock-change method calculates the C stocks in starting and ending inventories and estimates the differences in C stocks between the two points in time (Kurz et al. 2016). Furthermore, the CCT model is a fully empirical model based on field sampled measurements, whereas CBM is a hybrid model that uses empirical data from inventories (yield curves, forest attributes), but also employs process simulation to model dead organic matter and soil carbon pool dynamics (Kurz et al. 2009). Although InTEC also uses a hybrid approach, the modeling approach is even more process-based as it incorporates processes to model soil and nutrient dynamics, annual photosynthesis, hydrological dynamics, and nitrogen mineralization (Chen et al. 2000). Given these differences in forest area, datasets, and modeling approaches, some variation in C stock estimates is to be expected, but given that results of the models compare reasonably well lends validation to the use of the Canadian model for the U.S.

***Figure S8.*** *Comparison of total C stocks (Mg C ha-1) modeled using the Carbon Budget Model versus the Carbon Calculation Tool (Smith et al. 2010) and Integrated Terrestrial Ecosystem Carbon model (Dugan et al. 2017) for (a) all ownerships, (b) public, (c) Forest Service, and (d) private lands in the Northern Wisconsin study site.*

## 2.2 Climate and atmospheric effects

***Figure S9.*** *Accumulated carbon stock density (Mg C ha-1), calculated by consecutively summing the annual changes in C stock density due to individual disturbance\aging and non-disturbance factors and all factors combined from 1950-2010 excluding C accumulated pre-1950 for (a) Francis Marion and Sumter National Forests in the South Carolina study area and (b) Chequamegon-Nicolet National Forest in the Northern Wisconsin study area.*


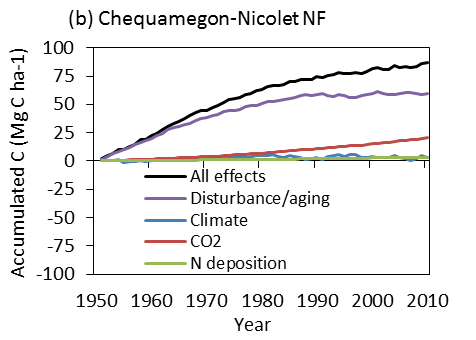

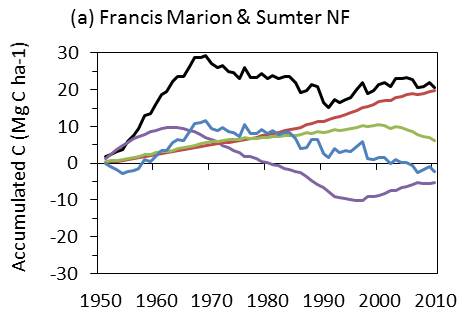


We also reported the potential impacts of atmospheric composition and climate on forest C dynamics in our study sites based on a related study (Dugan et al. 2017) because the ecosystem model used for this study does not include such analyses. Dugan et al. (2017) utilized the InTEC model to evaluate the relative effects of various disturbance (fires, harvests, insects, and aging/regrowth) and non-disturbance factors (climate, atmospheric CO_2_ concentrations, and nitrogen deposition) on C accumulation since 1950 within U.S. National Forests. While this current study expands to the public and private lands outside of the National Forests within our study area boundaries, the effects of broad-scale environmental drivers like climate on average annual C accumulation would be reasonably similar across the broader landscapes.

In both study areas, the effects of environmental factors on C accumulation have been significant since the 1950s (Fig. S10). In South Carolina’s Francis Marion and Sumter National Forest, the positive effects of CO_2_ fertilization and nitrogen deposition were strong enough to offset more recent C declines due to disturbances/aging and climate. Also in both study areas, the positive impacts of CO_2_ fertilization were generally greater than other environmental factors. Despite high levels of nitrogen deposition in northern Wisconsin, the effects of nitrogen deposition on C accumulation were relatively small as compared to South Carolina, which may be due to nitrogen saturation which has been found in Northeastern forests (Aber et al. 1998). Model results here and elsewhere (Dugan et al. 2017, Forkel et al. 2016) show the important role of atmospheric composition on forest C accumulation. Given this, the baseline projection in this analysis may underestimate carbon accumulation by assuming constant atmospheric CO_2_ concentrations throughout the historical and projection periods.

## 2.3 Hurricane mitigation scenario results

| **2030** |  |
| --- | --- |
| **2050** |  |

*F****igure S10.*** *Cumulative mitigation by component in 2030 and 2050 for the coastal South Carolina hurricane scenarios.*

# **3.0 Supplementary Materials References**

Aber J, McDowell W, Nadelhoffer K, Magill A, Berntson G, Kamakea M, McNulty S, Currie W, Rustad L, Fernandez I 1998 Nitrogen Saturation in Temperate Forest Ecosystems. *BioScience* **48** 921-934

Birdsey R A 1992 Carbon storage and accumulation in United States forest ecosystems. Gen. Tech. Rep. WO-59. Washington D.C.: U.S. Department of Agriculture, Forest Service, Washington Office. 51p.

Boudewyn P A, Song X, Magnussen S and Gillis M D 2007 Model-based, volume-to-biomass conversion for forested and vegetated land in Canada. Nat. Resour. Can., Can. For. Serv., Pac. For. Cent., Victoria, BC. Inf. Rep. BC-X-411.

Chen, J., Ter-Mikaelian, M. T., Yang, H., & Colombo, S. J. (2018). Assessing the greenhouse gas effects of harvested wood products manufactured from managed forests in Canada. *Forestry: An International Journal of Forest Research*, *91*(2), 193–205.

Chen, W., J.M. Chen, and J. Cihlar (2000). Integrated terrestrial ecosystem carbon-budget model based on changes in disturbance, climate, and atmospheric chemistry. Ecological Modelling, 135: 55-79.

Commission for Environmental Cooperation 2015 Integrated Modeling and Assessment of Forest Carbon Dynamics: Tools for monitoring, reporting and projecting forest greenhouse gas emissions. Montreal, Canada: *Commission for Environmental Cooperation*. 120 pp.

Coops N C, Wulder M A, White J C: Identifying and describing forest disturbance and spatial pattern: data selection issues and methodological implications; 2006. p 31–62.

Crookston N L and Dixon G E 2005 The forest vegetation simulator: A review of its structure, content, and applications. *Computers and Electronics in Agriculture* **49** 60–80

Dugan A J, Birdsey R, Healey S P, Pan Y, Zhang F, Mo G, Chen J, Woodall C, Hernandez AJ, McCullough K, McCarter J B, Raymond C L and Dante-Wood K 2017 Forest Sector Carbon Analyses Support Land Management Planning and Projects: Assessing the Influence of Anthropogenic and Natural Factors *Climatic Change* **144** 207-220

Eidenshink J, Schwind B, Brewer K, Zhu Z, Quayle B, Howard S 2007 A project for monitoring trends in burn severity *Fire Ecology* **3** 3–21

Earth Science Data and Information System (ESDIS) 2015 Fire Information for Resource Management System (FIRMS) *National Aeronautics and Space Administration* <https://earthdata.nasa.gov/earth-observation-data/near-real-time/firms>.

Forkel M, Carvalhais N, Rödenbeck C, Keeling R, Heimann M, Thonicke K, Zaehle S, Reichstein M (2016) Enhanced seasonal CO2 exchange caused by amplified plant productivity in northern ecosystems. Science 351: 696-699.

Fry J A, Coan M J, Homer C G, Meyer D K and Wickham J D 2009 [Completion of the National Land Cover Database (NLCD) 1992-2001 Land Cover Change Retrofit product](http://pubs.usgs.gov/of/2008/1379/): *U.S. Geological Survey Open-File Report* **1379** 18 p

Goeking, S. A. 2015. Disentangling forest change from forest inventory change: A case study from the US Interior West. Journal of Forestry. 113(5): 475-483.

Goward S N, Huang C, Zhao F, Schleeweis K, Rishmawi K, Lindsey M, Dungan J L and Michaelis A 2016 NACP NAFD Project: Forest Disturbance History from Landsat, 1986-2010. *ORNL DAAC*, Oak Ridge, Tennessee, USA

Haugen D E 2013 Wisconsin timber industry: an assessment of timber product output and use, 2008. Resour. Bull. NRS-78. Newtown Square, PA: *US Department of Agriculture* F*orest Service* *Northern Research Station* 110 p

Healey, S.P., Urbanski, S.P., Patterson, P.L. & Garrard, C. 2014. A Framework for Simulating Map Error in Ecosystem Models. Remote Sensing of Environment, 150, 207 – 217.

Healey, S. P., Cohen, W. B., Yang, Z., Kenneth Brewer, C., Brooks, E. B., Gorelick, N., … Zhu, Z. 2018. Mapping forest change using stacked generalization: An ensemble approach. Remote Sensing of Environment, *204*, 717–728.

Homer C, Dewitz J, Fry J, Coan M, Hossain N, Larson C, Herold N, McKerrow A, VanDriel, J N, and Wickham J 2007 [Completion of the 2001 National Land Cover Database for the Conterminous United States](http://www.asprs.org/a/publications/pers/2007journal/april/highlight.pdf) *Photogrammetric Engineering and Remote Sensing* **73** 337-341

Homer C G, Dewitz J A, Yang L, Jin S, Danielson P, Xian G, Coulston J, Herold N D, Wickham J D and Megown K 2015 [Completion of the 2011 National Land Cover Database for the conterminous United States-Representing a decade of land cover change information](http://bit.ly/1K7WjO3) *Photogrammetric Engineering and Remote Sensing* **81** 345-354

Houghton RA, Nassikas AA. 2017. Global and regional fluxes of carbon from land use and land cover change 1850-2015. *Global Biogeochemical Cycles* **31** 456-472.

Howard J L and Jones K C 2016 U.S. Timber production, trade, consumption, and price statistics, 1965-2013. Research Paper, FPL-RP-679. Madison, WI. *US Department of Agriculture Forest Service Forest Products Laboratory* 100 p

Huang C, Goward S N, Masek J G, Thomas N, Zhu Z, Vogelmann J E 2010 An automated approach for reconstructing recent forest disturbance history using dense Landsat time series stacks *Remote Sensing of Environment* **114** 183-198

IPCC 2006 Generic methodologies applicable to multiple land-use categories. IPCC Guidelines for National Greenhouse Gas Inventories, Ed. by S. Eggleston, L Buendia, K. Miwa, T. Ngara and K. Tanabe. Hayama, Japan: Intergovernmental Panel on Climate Change (IPCC) 1–59

IPCC 2013 Revised supplementary methods and good practice guidance arising from the Kyoto Protocol, *Institute for Global Environmental Scenarios*, Kanagawa, Japan

Johnson E W and Wittwer D 2008 Aerial detection surveys in the United States *Australian Forestry* **71** 212-215

Johnson J M F, Reicosky D C, Allmaras R R, Sauer T J, Venterea R T, Dell C J 2005 Greenhouse gas contributions and mitigation potential of agriculture in the central USA. *Soil & Tillage Research* **83** 73-94

Kull S, Rampley G, Morken S, Metsaranta J, Neilson E, Kurz W 2011 Operational-scale carbon budget model of the Canadian forest sector (CBM-CFS3) version 1.2: user’s guide. 2011. Edmonton, AB: *Canadian Forest Service.*

Kurz W A 2010 An ecosystem context for global gross forest cover loss estimates *PNAS* **107** 9025-9026

Kurz WA, Apps MJ, Comeau, PG, Trofymow JA 1996. The carbon budget of British Columbia’s forests: 1920 to 1989. Preliminary analysis and recommendations for refinements. Canada–British Columbia Partnership Agreement on Forest Resources Development: FRDA II. Nat. Resour. Can., Can. For. Serv., Pac. For. Cent.; BC Minist. For., Res. Br., Victoria, BC. FRDA Rep. 261. 62 p.

Kurz W, Birdsey R, Mascorro V, Greenberg D, Dai Z, Olguín M, Colditz R 2016 Integrated Modeling and Assessment of North American Forest Carbon Dynamics Technical Report: Tools for monitoring, reporting and projecting forest greenhouse gas emissions and removals. Montreal, Canada: *Commission for Environmental Cooperation* 120 pp

Kurz W A, Dymond C C, White T M, Stinson G, Shaw C H, Rampley G J, Smyth C, Simpson B N, Neilson E T, Trofymow J A, Metsaranta J and Apps M J 2009 CBM-CFS3: A model of carbon-dynamics in forestry and landuse change implementing IPCC standards *Ecol. Model*. **220** 480–504

Li, Z.; Kurz, W.A.; Apps, M.J.; Beukema, S.J. 2003. Belowground biomass dynamics in the Carbon Budget

Model of the Canadian Forest Sector: recent improvements and implications for the estimation of NPP and NEP. Can. J. For. Res. 33:126–136.

Mills, JR and Kincaid, JC.1992. The Aggregate Timberland Assessment System-ATLAS: A comprehensive timber projection model Gen. Tech. Rep. PNW-GTR-281, US Department of Agriculture, Forest Service, Pacific Northwest Research Station, Portland, OR, p. 160

Mascorro, V. S., Coops, N. C., Kurz, W. A., & Olguín, M. (2015). Choice of satellite imagery and attribution of changes to disturbance type strongly affects forest carbon balance estimates. Carbon Balance and Management, 10. doi.org/10.1186/s13021-015-0041-6

National Inventory Report (NIR 2014) 1990–2012: Greenhouse Gas Sources and Sinks in Canada, Environment Canada, 2014.

O’Connell, Barbara M.; Conkling, Barbara L.; Wilson, Andrea M.; Burrill, Elizabeth A.; Turner, Jeffery A.; Pugh, Scott A.; Christiansen, Glenn; Ridley, Ted; Menlove, James. 2017. The Forest Inventory and Analysis Database: Database description and user guide version 7.0 for Phase 2. U.S. Department of Agriculture, Forest Service. 830 p. [Online]. Available at http://www.fia.fs.fed.us/library/database‐documentation/

Oswalt, Sonja N.; Smith, W. Brad; Miles, Patrick D.; Pugh, Scott A. 2014. Forest Resources of the United States, 2012: a technical document supporting the Forest Service 2015 update of the RPA Assessment.Gen. Tech. Rep. WO-91. Washington, DC: U.S. Department of Agriculture, Forest Service, Washington Office. 218 p.

Peckham, S. D., S. T. Gower, C. H. Perry, B. T. Wilson, and K. M. Stueve. 2013. Modeling harvest and biomass removal effects on the forest carbon balance of the Midwest, USA. *Environmental Science & Policy* **25**:22–35.

Pilli, R, Grassi G, Kurz WA, Smyth CE, Blujdea V. 2013. Application of the CBM-CFS3 model to estimate Italy’s forest carbon budget, 1995–2020. Ecological Modelling 266:144-171.

Potter, C. S. (1999). Terrestrial Biomass and the Effects of Deforestation on the Global Carbon Cycle: Results from a model of primary production using satellite observations. *BioScience* **49** 769–778

Potter, R. S., Andrews, S., Atwood, J.D., Kellogg, R.L., Lemunyon, J., Norfleet, L., Oman, D. 2006. Model Simulation of Soil Loss, Nutrient Loss, and Change in Soil Organic Carbon Associated with Crop Production. US Department of Agriculture, Natural Resources Conservation Service, Conservation Effects Assessment Project

PRISM Climate Group, *Oregon State University* http://prism.oregonstate.edu created 10 November 2016

Raymond, C. L., Healey, S., Peduzzi, A., Patterson, P. 2015. Representative regional models of post-disturbance forest carbon accumulation: Integrating inventory data and a growth and yield model. *Forest Ecology and Management* 336: 21-34.

Ruefenacht B, Finco MV, Nelson MD, Czaplewski R, Helmer EH, Blackard JA, Holden GR, Lister AJ, Salajanu D, Weyermann D, Winterberger K (2008) Conterminous US and Alaska forest type mapping using forest inventory and analysis data. Photogrammetric Engineering & Remote Sensing 74: 1379–1388.

Running, S. W., & Gower, S. T. (1991). FOREST-BGC, A general model of forest ecosystem processes for regional applications. II. Dynamic carbon allocation and nitrogen budgets. *Tree Physiology* **9** 147–160

Sathre R and O’Connor J. 2010. Meta-analysis of greenhouse gas displacement factors of wood product substitution. Environmental Science & Policy, 13, 104–114.

Skog K E 2008 Sequestration of carbon in harvested wood products for the United States [***Forest Products Journal***](http://search.proquest.com/pubidlinkhandler/sng/pubtitle/Forest+Products+Journal/$N/25222/DocView/214611158/fulltext/6E8A9BD66DE04CB6PQ/1?accountid=28147) **58** 56-72

Sheffield RM and Thompson MT 1992 Hurricane Hugo: Effects on South Carolina’s Forest Resource. Research Paper **SE-284** *USDA Forest Service Southeastern Forest Experiment Station* Ashville, NC

Smith J E, Heath L S and Nichols M C 2010 US Forest Carbon Calculation Tool: forest-land carbon stocks and net annual stock change. Revised. Gen. Tech. Rep. NRS-13. Newtown Square, PA: *US Department of Agriculture Forest Service Northern Research Station* 34 p.

Smyth C E, Kurz WA, Rampley G, Lemprière T C, Schwab O 2016 Climate change mitigation potential of local use of harvest residues for bioenergy in Canada *GCB Bioenergy* **9** 817-832

Smyth C E, Rampley G, Lemprière T C, Schwab O, Kurz W A 2017 Estimating product and energy substitution benefits in national-scale mitigation analyses for Canada *GCB Bioenergy* **9** 1071-1084

Smyth C E, Stinson G, Neilson E, Lemprière, T C, Hafer M, Rampley G J, and Kurz W A 2014 Quantifying the biophysical climate change mitigation potential of Canada’s forest sector. Biogeosciences 3515-3529.

 Spalding D. The role of forests in global carbon budgeting. Forests an. In: Tyrrell ML, Ashton MS, Spalding D, Gentry B, Editors. Yale School of Forestry & Environmental Studies; 2009. p 223–53.

Suter, F., Steubing, B., & Hellweg, S. (2017). Life cycle impacts and benefits of wood along the value chain: The case of Switzerland. Journal of Industrial Ecology 21: 874-886.

Trottier-Picard A, Thiffault E, Desrochers A., Paré D, Thiffault N and Messier C 2014 Amounts of logging residues affect planting microsites: A manipulative study across northern forest ecosystems *For. Ecol. Manag*. **312** 203–215

Turner MG 2010 Disturbance and landscape dynamics in a changing world *Ecology* **91** 2833-2849

USDA Forest Service 2016 Forest Inventory and Analysis National Program: FIA Data Mart. U.S. Department of Agriculture Forest Service. Washington, DC. Available online at <http://apps.fs.fed.us/fiadb-downloads/datamart.html> >. Accessed 1 October 2016.

US Department of Agriculture Forest Service [Nd] Timber Product Output (TPO) Reports. Knoxville, TN: *US Department of Agriculture Forest Service Southern Research Station* http://srsfia2.fs.fed.us/php/tpo_2009/tpo_rpa_int1.php. [Date accessed: November 2016]

Van Deusen P C and Heath L S 2010 Weighted analysis methods for mapped plot forest inventory data: Tables, regressions, maps and graphs *Forest Ecol. Manage* **260** 1607-1612

Walmsley J D and Godbold D L 2010 Stump Harvesting for Bioenergy – A Review of the Environmental Impacts. Forestry (Lond) **83** 17-38

Werner F, Taverna R, Hofer P, Thürig E and Kaufmann E 2010 National and global greenhouse gas dynamics of different forest management and wood use scenarios: a model-based assessment. *Environmental Science & Policy* **13** 72–85

Woodall C W, Heath L S, Domke G M and Nichols M C 2011 Methods and equations for estimating aboveground volume, biomass, and carbon for trees in the U.S. forest inventory, 2010. Gen. Tech. Rep. NRS-88. Newtown Square, PA: *US Department of Agriculture Forest Service Northern Research Station* 30p

Wulder M A and Coops N C 2014 Satellites: Make Earth observations open access. *Nature* **513** 30-31

Zhang, F., J.M. Chen, Y. Pan, R.A. Birdsey, S. Shen, W. Ju, and L. He. 2012. Attributing carbon changes in conterminous US Forests to disturbance and non-disturbance factors from 1901 to 2010 *Journal of Geophysical Research: Biogeosciences* **117** (G02021) 1–18. doi:10.1029/2011JG001930.
